# Pre-harvest heat stress affects rocket salad leaf transcription and metabolism at harvest and after chilled postharvest storage

**DOI:** 10.1093/aob/mcaf243

**Published:** 2025-11-13

**Authors:** Lama M N Alotaibi, Charlotte Wilson, Ashley Baldwin, Kashia Dias, Ella Whiteford, Elenia Parkes, Corin Mylett, Jonathan Galbusera, Angela Marchbank, Manfred Beckmann, Natasha D Spadafora, Carsten T Müller, Sarah Christofides, Hilary J Rogers

**Affiliations:** Department of Biology, College of Science, Imam Abdulrahman Bin Faisal University, P.O. Box 1982, Dammam 31441, Saudi Arabia; School of Biosciences, Cardiff University, Cardiff CF10 3AX, UK; School of Biosciences, Cardiff University, Cardiff CF10 3AX, UK; School of Biosciences, Cardiff University, Cardiff CF10 3AX, UK; School of Biosciences, Cardiff University, Cardiff CF10 3AX, UK; School of Biosciences, Cardiff University, Cardiff CF10 3AX, UK; School of Biosciences, Cardiff University, Cardiff CF10 3AX, UK; School of Biosciences, Cardiff University, Cardiff CF10 3AX, UK; School of Biosciences, Cardiff University, Cardiff CF10 3AX, UK; High Resolution Metabolomics Laboratory, Aberystwyth University, Aberystwyth SY23 3DA, UK; School of Biosciences, Cardiff University, Cardiff CF10 3AX, UK; Department of Environmental Sciences and Prevention, University of Ferrara, Ferrara 44121, Italy; School of Biosciences, Cardiff University, Cardiff CF10 3AX, UK; School of Biosciences, Cardiff University, Cardiff CF10 3AX, UK; School of Biosciences, Cardiff University, Cardiff CF10 3AX, UK

**Keywords:** *Diplotaxis tenuifolia*, heat stress, metabolome, postharvest storage, rocket salad, transcriptome, volatilome

## Abstract

**Background and Aims:**

Climate change is resulting in increasingly variable weather patterns with spikes of high temperatures adversely affecting crop production. Here the effect of elevated temperature just before harvest was investigated in wild rocket (*Diplotaxis tenuifolia*), a popular brassicaceous salad. The key aim was to investigate how pre-harvest stress affects postharvest responses.

**Methods:**

Mature rocket plants were subjected to 3 d of elevated daytime temperature (35 °C) before harvest. Leaves were then stored at 6 °C to mimic postharvest supply chain conditions. Physiological data were collected at harvest and after 7, 14 and 21 d of storage. Volatile organic compounds (VOCs) were analysed by gas chromatography–mass spectrometry, changes in metabolite profiles were analysed through flow injection electrospray high-resolution mass spectroscopy, and gene expression was assessed by RNAseq and real-time PCR.

**Key Results:**

Transcriptomic analysis showed a mild heat stress signature affecting both metabolic and regulatory genes, including those related to hormone signalling. Models for effects on circadian clock genes and regulation of cold/dark stress responses are derived based on *Arabidopsis thaliana* pathways. After 7 d of storage, there were also significant effects of the pre-harvest heat stress on leaf VOC profiles, with distinct patterns compared with those at harvest. The metabolome was also affected after 7 d of storage, with specific effects on several lipid classes, amino acids and sugars. However, the direction of gene expression changes did not always match effects on VOCs. After 21 d of storage, pre-harvest heat stress adversely affected chlorophyll content and photosynthetic capacity, promoted ion leakage, and resulted in increased stomatal closure.

**Conclusions:**

Cold storage affects the physiology, gene expression and metabolome of rocket leaves and these effects are perturbed by exposure to heat stress before harvest.

## INTRODUCTION

*Diplotaxis tenuifolia*, known as perennial wall rocket, or wild rocket, is a widely cultivated crop species, with commercial importance in ready-to-eat salads (Villani *et al.*, 2023). However, rocket salad has a short shelf life of only 12–14 d; 7 d shelf-life is typical in most retail supply chains to ensure microbial safety and the desired sensory and physiochemical qualities (Danza *et al.*, 2015; Torres-Sánchez *et al.*, 2020). To maintain quality, rocket salad is typically stored and transported at 2–5 °C, while higher temperatures are experienced on the retail shelf (Watada *et al.*, 1996).

Crops are subjected to multiple dynamic stresses during growth in the field. These stresses are increasingly variable and severe as a result of climatic changes (Saqib *et al*., 2022). Optimal growth of *D. tenuifolia* is attained between 20 and 30 °C (Jasper *et al.*, 2020). Exposure of *D. tenuifolia* to higher temperatures (30–44 °C) has a significant effect on biomass and chlorophyll content (Cacini *et al.*, 2024). Severe heat stress directly affects cellular function, and can lead to cell damage and cell death, while milder stress results in thermomorphogenesis, with effects on plant morphology (Jagadish *et al.*, 2021). In *Arabidopsis*, more severe heat stress activates a transcriptional network, with *HEAT SHOCK FACTOR*A1 (*HsfA1*) genes having a central role (Ohama *et al.*, 2017). These in turn activate *HsfA2*, *HsfA7* and *HsfB*, which are DREB2a transcription factors. Together with a trimer of *NF-YA2*, *NF-YB3* and *DPB3-1* they switch on the expression of a wide range of chaperones and enzymes that provide protective roles for the cell, including heat shock proteins (Hsps) (Swindell *et al.*, 2007). The transcription factors *PIF4* and *PIF5* appear to play a key role in thermomorphogenetic responses to milder heat stress in *Arabidopsis*. PIF4 expression is upregulated in response to elevated temperature (Koini *et al.*, 2009) and along with PIF5 it regulates phytohormone levels and a network of downstream genes (Casal and Balasubramanian, 2019). *NAC19*, *IAA29* and *SAG113* have been identified as direct targets of PIF4/5 (Li *et al.*, 2021) and these genes also play an important role in heat stress-induced leaf senescence. Heat stress responses are mediated through several different phytohormones. *Arabidopsis* mutants of ABA biosynthesis and response genes are defective in heat stress tolerance (Larkindale *et al.*, 2005), and ABA content appears to increase transiently within the first 2 h of heat treatment in *Arabidopsis* (Dobrá *et al.*, 2015). Auxin is important in thermomorphogenesis and *PIF4* regulates auxin levels at high temperature, activating the expression of genes encoding SAUR proteins (Franklin *et al.*, 2011). In *Arabidopsis*, up to 50 % of the temperature-responsive genes have a rhythmic pattern (Covington *et al.*, 2008). PIF4/5 are key components of the circadian clock, targeted for degradation by phytochromes under light, and stabilized in the dark (Leivar *et al.*, 2012). The core components of the circadian clock are CCA1, LHY, PRR7 and PRR9, which are maximally active in the morning. During the day PIF4 and 5 are maximally expressed in the early morning, and repressed at dusk by ELF3, ELF4 and LUX (Herrero *et al.*, 2012).

Pre-harvest growth conditions can affect leaf colour and texture postharvest (Edelenbos *et al.*, 2017). Pre-harvest stresses, including salinity, exposure of roots to high temperature and nitrogen starvation, were shown to affect global gene expression in *D. tenuifolia* (Cavaiuolo *et al.*, 2017) when assessed 24 h after the stress treatment, including changes in transcription factors, genes related to plant growth regulator signalling, autophagy and senescence.

Postharvest, leaves experience a combined stress treatment comprising mechanical damage during processing, cold stress as well as dark or low light, and dehydration (Pirovani *et al.*, 1998). These stresses have detectable effects on metabolism and on the profile of volatile organic compounds (VOCs) comprising the aroma, which change progressively throughout the storage period (Luca *et al.*, 2016; Spadafora *et al.*, 2016). VOC families detected in the headspace of rocket salad include alcohols, aromatic compounds, esters, terpenes and, importantly, isothiocyanates, with compounds such as dimethyl sulphide and dimethyl disulphide rising towards the end of shelf life (Luca *et al.*, 2016; Spadafora *et al.*, 2018).

Mechanical damage initiates a wounding response characterized by the release of green leaf volatiles (GLVs; ul Hassan *et al.*, 2015), important to the plant as signalling molecules. These comprise 6-carbon molecules, including the aldehyde hexanal, the alcohol hexen-1-ol and its ester hexenyl acetate. They are derived from the oxylipin pathway through the action of lipoxygenases (LOXs), lipases and hydroxyperoxide lyase enzymes on membrane lipids, including phospholipids and glycerolipids. This is the same pathway that leads to the production of the phytohormone jasmonic acid (JA), whose production is also triggered by wounding (León *et al.*, 2001).

Dark-induced senescence has been extensively studied in *Arabidopsis*, with a growing understanding of the regulatory pathways (Liebsch and Keech, 2016). Darkness inactivates phytochromes PHYA and PHYB, which under light conditions promote the degradation of PIF4/5. PIF4/5 play an important role in dark-induced senescence by activating the transcription factors *ETHYLENE INSENSITIVE 3* (*EIN3*), *ABSCISIC ACID INSENSITIVE 5* (*ABI5*) and *ENHANCED EM LEVELS* (*EEL*) as well as ethylene biosynthesis genes (*ACS*). These transcription factors in turn activate the NAC transcription factor *ORE1* (*ANAC092*), which is also activated by ATAF1 (ANAC002). ORE1 activates the expression of *SGR1* and *NYC1* involved in chlorophyll degradation as well as senescence-associated genes (SAGs) such as *SAG12*, which is involved in macromolecule breakdown. Dark-induced senescence thus ultimately leads to loss of photosynthetic capacity, chloroplast disassembly and the death of the leaf through a process of stress-induced senescence. It appears that *PIF4/PIF5* act upstream of ABA signalling in dark-induced senescence, while ethylene signalling may be downstream of ABA but also regulated by PIF4/PIF5 semi-independently (Ueda *et al.*, 2020). Other NAC transcription factors and WRKY transcription factors are also involved in dark-induced senescence: *AtNAP* (*ANAC029*) is upregulated by ABA, which rises during senescence and during dehydration stress and targets directly *SAG113*. This gene encodes a protein phosphatase 2C (PP2C) that inhibits stomatal closure during senescence, enabling oxygen entry for respiration (Zhang and Gan, 2012). ABA also induces a set of stress-responsive NAC transcription factors named collectively as SNAC-As and comprise *ANAC055*, *ANAC019*, *ANAC072/RD26*, *ANAC002/ATAF1*, *ANAC081/ATAF2*, *ANAC102* and *ANAC032* (Takasaki *et al.*, 2015).

Exposure of leaves to prolonged chilling at temperatures above freezing, as experienced during postharvest storage, also elicits complex transcriptional and metabolic changes (Kidokoro *et al.*, 2022) involving over 2000 genes in *Arabidopsis* (Park *et al.*, 2015). DREB1/CBF transcription factors act as master regulators. They are activated by CAMTA3, CAMTA5, RVE4 and RVE8, while CCA1, LHY and PIF4/5 repress them. Downstream of DREB1/CBF are a large number of cold-regulated (COR) genes, which encode a range of enzymes and proteins involved in cold tolerance (Kidokoro *et al.*, 2022). In parallel, *RVE4/8* and *LHY* also activate *COR* genes independently of *DREB1/CBF*. Cold also activates other *DREB1*-independent genes, such as *ZAT12*, which also activate the *COR* genes. An increase in ABA is an important component of cold stress responses inducing the transcription factor MYB96, which integrates cold and ABA signalling pathways. Brassinosteroids promote cold stress responses, by affecting redox homeostasis (Kim *et al.*, 2012). JA signalling via the degradation of JASMONATE ZIM DOMAIN (JAZ) proteins induces cold responses, and JAZ1 and JAZ4 are key players (Hu *et al.*, 2013). At a cellular level, cold stress alters membrane fluidity and composition. For example, after 1 week at 2 °C the membrane lipids contained more phospholipids (Uemura *et al.* (1995). Sphingolipid composition also changes in response to cold in several species (Huby *et al.*, 2020) and *Arabidopsis* double mutants of *SLD1* and *SLD2* genes, which are involved in sphingolipid desaturation, are hypersensitive to cold (Chen *et al.*, 2012). Cold also activates AtBI-1, which is involved in both suppressing cell death and in sphingolipid biosynthesis through its interaction with ceramide-modifying enzymes (SLD1, FAH1, SBH2 and ADS2) (Nagano *et al.*, 2014). Indeed, sphingolipids may also have a signalling function in response to stress and interact with phytohormone signalling (Huby *et al.*, 2020). Downstream of the regulatory network, synthesis of osmoprotectants, including sugars and amino acids, is activated as a protective mechanism during cold stress. Proline accumulation is particularly important, acting both as an osmoprotectant and an antioxidant (Meena *et al.*, 2019). Other amino acids, such as leucine, valine and isoleucine, also often increase under stress, although their role is less clear (Hildebrandt, 2018).

Relatively few studies have been focused on combined stresses, although this is a growing field of research as it mirrors more closely the complexity of the field pre- and postharvest. In *Arabidopsis* combined cold and dark treatment was seen to result in opposing signals. The dark treatment induced senescence, while the cold delayed it due to the reduction in metabolic activity (Panigrahy *et al.*, 2022). Senescence under these conditions was promoted by ABA while it was delayed by brassinosteroid signalling.

In this study, we assess the effects of stresses experienced by rocket salad leaves during pre-harvest mild heat stress followed by postharvest chilled dark storage. To gain a better understanding of different patterns of change we integrate volatilomic, metabolomic and transcriptomic analysis with physiological changes. This enables us to identify changes in phytohormone responses and link them to changes in lipid metabolism and VOC biosynthesis. The close phylogenetic relationship to *Arabidopsis* allows us to propose a model for changes in expression of key transcription factors and how these result in physiological changes.

## MATERIALS AND METHODS

### Plant growth and treatments

Seeds of *Diplotaxis tenuifolia* cv. ‘Frastagliata’ were sown in pots (5 cm × 6.5 cm) filled with a mixture of compost (Levington Advance, ICL Professional Horticulture) and sand (Vitax) (3:1), with eight seedlings per pot. Plants were grown under controlled conditions (22 °C day, 18 °C night; 155 μmol m^−2^ s^1^; 16 h of light) to mature leaf stage (25–30 d). Heat treatment was applied for 72 h stress (35 °C day, 25 °C night) to at least 18 pots with 18 pots as control. Leaves were removed from the plants with a sharp blade and stored at 6 °C for up to 21 d in closed containers in an incubator (IL-21 Jeiotech, South Korea).

### Physiological analyses

All physiological assessments were conducted between 1000 and 1100 h on each time point day to reduce the chance of the circadian rhythm of the rocket salad influencing the results (Ruiz de Larrinaga *et al.*, 2019).

Ion leakage was measured using a modified method from Jalil *et al.* (2017). A sterile cork borer was used to cut eight 8-mm leaf discs from either side of the central vein of randomly selected leaves, one for each biological replicate. Discs were floated in 20 mL of distilled water for 2 h, then transferred to 50-mL centrifuge tubes containing 20 mL of fresh distilled water and rotated for 24 h at room temperature using a RotoFlex™ Plus Tube Rotator. Conductivity was measured using a Hanna HI-2003 Edge conductivity meter. To obtain the total conductivity, the leaf discs were heated for 1 h at 86 °C and ion leakage for each leaf is expressed as relative conductivity.

Chlorophyll concentration was estimated using a SPAD-502 Plus portable chlorophyll meter (Minolta, Osaka, Japan), as in Uddling *et al.* (2007) using the mean of three readings from each leaf and three biological replicates. Photosynthetic capacity was measured using a MINI-PAM-II (pulse-amplitude modulation) photosynthesis yield analyser (Walz, Germany) as in Motohashi and Myouga (2015), measuring *F*_v_/*F*_m_. Three measurements were taken from each leaf for each biological replicate.

The degree of stomatal closure was measured as in Pathoumthong *et al.* (2023) using a thin layer of clear nail polish painted onto the underside of each leaf. Once dry, this was removed using a scalpel and imaged using a light microscope. Images were captured using GT^©^ software, with ImageJ used to measure the width and length of each stoma. For three leaves (biological replicates), 30–80 stomata were measured. Results are reported as the ratio of width to length.

Stomatal conductance (combined CO_2_ and H_2_O) vapour flux was measured using an SC-1 Leaf Porometer (METER, München). Three measurements (technical replicates) were taken per leaf, avoiding the primary vein, and averaged. Three leaves were measured as three biological replicates for each treatment and time point.

### VOC analyses

VOC sampling and analysis was adapted from Spadafora *et al.* (2019). Rocket leaves (3 g) from each treatment were placed inside a Nalophan bag (25 cm × 30 cm, Lakeland Ltd, Windermere, UK) and filled with ambient air (with three biological replicates for repetition 1 and four for repetition 2). The bag was sealed and stored at room temperature for 1 h to equilibrate the headspace. After sealing the bag, the leaves were given a light rub to release the VOCs. An empty bag with no leaves (filled with ambient air) was used as a control sample. Headspace (1000 mL) was collected with an Easy-VOC manual pump (Markes International Ltd, Llantrisant, UK) onto SafeLok™ thermodesorption (TD) tubes (Tenax TA & Sulficarb, Markes International Ltd, Llantrisant, UK). As retention standards, 1 μL C8-C20 alkane standard (Sigma–Aldrich, St Louis, MO, USA) was loaded directly onto TD tubes. The TD100 thermal desorption system (Markes International Ltd., Llantrisant, UK) was used to desorb the tubes with the following settings: 5 min at 100 °C followed by 5 min at 280 °C, at a trap flow of 40 mL min^−1^; for trap desorption and transfer the settings were 20 °C s^−1^ to 300 °C, splitting the flow of 5 mL min^−1^ into GC (7890A; Agilent Technologies, Inc., Santa Clara, CA, USA). A constant flow of 1.5 mL min^−1^ helium was used to separate VOCs over 60 m, 0.32 mm ID, 0.5 μm MEGA-5-MS (MEGA Srl, Legnano, Italy), using the following temperature programme: initial temperature 40 °C for 2 min, 5 °C min^−1^ to 240 °C, then 20 °C min^−1^ to 300 °C and final hold for 2 min at 300 °C. A time-of-flight mass spectrometer (BenchTOF-dx, Markes International Ltd, Llantrisant, UK) was used to record the mass spectra from *m*/*z* 35–450. Data obtained from GC–MS were first processed using MSD ChemStation software (E.02.01.1177; Agilent Technologies, Santa Clara, CA, USA).

Data were deconvoluted and integrated using a custom retention-indexed mass spectral library with AMDIS (v2.72). MS spectra were searched against the NIST 2014 library (version 2.2g, 2014; Mikaia *et al.*, 2014). Compounds that scored >80 % in forward and backward match factor and those with a retention index (RI) close to the NIST database value (RI ±30) were included in the custom mass spectral library as putatively identified. Compounds that scored >80 % in forward and backwards and with no matching RI were included as chemical family, e.g. ‘alkane’, and compounds that with the same RI and the same mass spectrum in different samples but scores <80 % were added as ‘unknown’. The data obtained from different AMDIS files were combined using GC–MS Assignment Validator and Integrator (GAVIN v3.97) script in (MATLAB R2019b) to generate a peak table (Behrends *et al.*, 2011). Final data were generated following the adjustment of the size and position of the integration windows. Compounds that were not abundant in at least all replicates of one treatment compared with controls, and known contaminants (Supplementary Data Table S1) were excluded from the statistical analyses. VOC data have been deposited in the MetaboLights repository (Yurekten *et al.*, 2024).

### RNA extraction cDNA synthesis and real-time qPCR

For total RNA extraction, 1 g of randomly selected leaves (∼10–12 leaves) for each of three biological replicates was ground under liquid nitrogen in a mortar and pestle. From 100 mg of this ground leaf material RNA was extracted according to Spadafora *et al.* (2019) by using TRI reagent (Sigma, Dorset, UK) according to the manufacturer’s instructions. Final pellets were allowed to dry for 10–30 min then re-suspended in 50 μL nuclease-free water and stored at −80 °C. The quality of RNA was checked by NanoDrop spectrophotometry (Implen™ NanoPhotometer™ NP80) and by agarose gel electrophoresis.

For cDNA synthesis, residual genomic DNA was removed from the RNA (2 μg) using RQ1 DNase (Promega UK) and tested by PCR using translation elongation factor (EF1a) primers (Supplementary Data Table S2). cDNA synthesis was conducted using GoScript™ (Promega UK) with oligo(dT) priming, extension at 42 °C for 60 min and inactivation at 70 °C for 15 min.

Real-time quantitative PCR (qPCR) was performed in 20-μL reactions containing 10 μL SyberGreen Blue ready mix (PCR Biosystems), 3.2 μL nuclease-free water, 0.4 μL of the primers (forward and reverse) and 6 μL of cDNA. Dilution of the cDNA was adjusted based on the number of qPCR cycles (quantification cycle) (Bustin *et al.*, 2009) for the housekeeping gene (*EF1a*). Reactions were amplified in a LightCycler^®^ 96 with two technical replicates and three biological replicates. The qPCR thermal programme consisted of one preincubation cycle at 95 °C for 120 s followed by 35 cycles of three amplification steps (95 °C for 30 s, 55 °C or 60 °C for 30 s and 72 °C for 30 s). Post-cycling, a melting curve was also run on the samples to check primer specificity (95 °C for 60 s, 55 °C for 30 s, 95 °C for 1 s). The ΔΔCt method was used to analyse the data by comparison with *DtEF1a* (Livak and Schmittgen, 2001). Since the replicates were unpaired, pairwise differences were calculated for each replicate of the non-stressed control compared with all other replicates from any treatment. For each treatment group, the mean of these pairwise differences was then taken to represent the ΔΔCT.

### Transcriptome analysis

For transcriptomic analysis RNA was extracted using a RNeasy Mini Kit (Qiagen)). The quality of RNA samples was checked using a Qubit assay (Qubit RNA HS Assay Kit, Invitrogen) and then tested on RNA ScreenTape (Agilent Technologies) on a TapeStation 2200 (Agilent Technologies). Before sequencing, an Illumina RNA Prep with Enrichment (Illumina) kit was used for cDNA library preparation, and the quality was assessed using a D1000 ScreenTape (Agilent Technologies) on a TapeStation 2200 (Agilent Technologies). Paired-end sequencing was conducted on an Illumina NovaSeq 5000 for all samples.

Scripts used in the bioinformatic analysis are available at github.com/ecologysarah/RocketPostHarvest. The quality of RNA sequencing data was assessed using FastQC (Chen, 2023) and multiQC (Ewels *et al.*, 2016). FastP software (version 0.23.1) was used to remove adapter sequences and poor-quality bases/sequences from the reads. A *de novo* assembly for all reads into a reference transcriptome was conducted using the Trinity software package (v2.6.6) (Grabherr *et al.*, 2011). Annotation of the reference transcriptome was done using EviGene software (version 2019.10, v4) (Gilbert, 2019), and assembly completeness was assessed with BUSCO (v4.0.6) (Seppey *et al.*, 2019). Read mapping was performed using RSEM (v1.3.3) (Li and Dewey, 2011) for transcript quantification and Bowtie2 (v2.4.1) (Langmead and Salzberg, 2012) for estimation of gene/isoform expression for each sample individually. EdgeR (Chen *et al.*, 2014) was used to compare the expression levels amongst treatments. Annotation of the assembled contigs was performed using BLASTX (Camacho *et al.*, 2009); https://blast.ncbi.nlm.nih.gov/Blast.cgi against *Arabidopsis thaliana* (TAIR peptide sequences). *Arabidopsis thaliana* gene identifiers were used for further Gene Ontology (GO) term enrichment, KEGG pathway analysis, functional profiling and transcription factor prediction. Functional analysis of DEGs used a cut-off of <0.001 adjusted *P*-value (FDR) and log_2_ fold change (log2 FC) higher than 1.4 and lower than −1.4. The web-based tool Venny 2.0 (Oliveros, 2007) was used to create Venn diagrams. The online GO term/KEGG pathway enrichment tool ShinyGO (v0.77) (Ge *et al.*, 2020) was used to identify enriched biological processes by comparing the frequency of particle GO terms in the complete and unique set of genes for each comparison with the expected frequency in the background. The background consisted of all the putative genes identified in the transcriptome assembly, which had a significant alignment with an *A. thaliana* gene. GO terms that did not reach statistical significance with the cut-off of adjusted *P*-value (FDR) > 0.001 were excluded. The web-based tool gProfiler (Raudvere *et al.*, 2019) was used to identify significantly overrepresented biological processes. KEGG pathway analysis was carried out online using the KEGG Mapper tool (Kanehisa and Goto, 2000). The web-based tool bioDBnet:db2db (Mudunuri *et al.*, 2009) was used for the conversion of *A. thaliana* codes to KEGG codes.

The web-based transcription factor (TF) prediction tool PlantRegMap/PlantTFDB (v5.0) (Tian *et al.*, 2020) was used to identify TFs with the best *A. thaliana* hit in the transcriptome.

### Metabolome analysis

Metabolites were extracted with chloroform/methanol/water (1:3:1) from 100 mg of fragmented leaves (eight replicates) stored at −80 °C. A single-run flow injection electrospray–high-resolution mass spectroscopy (FIE-HRMS) approach was used on an Exactive HCD mass analyser coupled to an Accela UHPLC system (Thermo-Scientific), which produced metabolite fingerprints in both positive and negative ion mode. Extract (60 μL) was directly injected into a flow of 100μL min^−1^ methanol: water (70:30, v/v). Ion intensities within the 50 and 1000 *m*/*z* range were measured at a resolution setting of 100 000 (at *m*/*z* 200), resulting in mass accuracies of 3 (±) ppm for 3.5 min. ESI source parameters adhered to manufacturer guidelines. Exported CDF files underwent mass alignment and centroid in MATLAB (v8.2.0, MathWorks). Mass spectra around the peak apex spectra were combined for each ion mode, generating intensity matrices. Subsequent log transformation of the intensity matrices prepared the data for statistical analysis. Random Forest in R package FIEms-pro (Enot *et al.*, 2008) was used for data extraction and feature selection. Compounds that were only in 2/3 replicates of one treatment class were removed from the data. The data were then normalized to the total ion content. Informative compounds identified by Random Forest analysis were searched against a plant metabolite database (https://dimedb.ibers.aber.ac.uk/search/mass) using the measured *m*/*z* ratio, ionization type, and any available ion type information for each compound (O’Shea *et al.*, 2018). Potential matches were then evaluated for their accuracy against the measured *m*/*z* and the possibility of their presence in plant tissue. Due to the fundamental uncertainty of this method, only the wider chemical class(es) of the suggested compounds are reported rather than specific compound names.

The same samples were also analysed using gas chromatography–time of flight mass spectrometry (GC-TOF-MS), identifying sugars and amino acids. A supernatant extract (5 μL) and 5, 20 and 30 μL of the carbohydrate standards were used. An internal standard (25 μL L-threo-tertbutylserine) was added and carbonyl moieties of metabolites were protected by methoximation using 10 μL 20 mg mL^−1^ solution of methoxyamine hydrochloride (Fluka) for 90 min at 30 °C. Treatment with 20 μL *N*-methyl-*N*-trimethylsilyl-trifluoroacetic acid (MSTFA, M and N) at 37 °C for 30 min was utilized to derivatize acidic protons within the sample into more volatile molecules. The derivatized sample (1 μL) was then injected splitlessly into a Leco Pegasus III GC-TOF-MS (St Joseph, USA), comprising a Focus autosampler (Anatune), and an Agilent 6890N gas chromatograph equipped with a DB5-MS column (20 m × 0.25 mm ID × 0.25 μm film), configured with precise temperature settings of 250 °C for the injector, 260 °C for the transfer line and 230 °C for the ion source. A constant helium flow of 1.4 mL min^−1^ was maintained. Following 1 min at 80 °C, an oven temperature gradient was applied with 30 °C min^−1^ to 330 °C, held at 330 °C for 3 min and cooled to 80 °C. Automated peak deconvolution and identification were accomplished by using ChromaTof software (Leco, St Joseph, USA), with subsequent peak alignment performed in MATLAB (v7.5.0, MathWorks). Metabolome data have been deposited in the MetaboLights repository (Yurekten *et al.*, 2024)

### Statistical analysis

VOC data were normalized as a percentage of the total area for that sample and square rooted. Analysis was conducted in R using Random Forest as implemented in the package RandomForest (Liaw and Wiener, 2002). The cut-off for mean decrease accuracy was selected on the basis of the magnitude of change, and tested iteratively to determine the minimum number of compounds that retained or improved discrimination compared with the full set. Physiological, gene expression and metabolite data were checked for normality of distribution and analysed using a two-way ANOVA followed by a Tukey (multiple comparisons test) or a Dunn test followed by a Kruskal–Wallis test as appropriate. Differences were considered statistically significant at a value of *P* ≤ 0.05.

## RESULTS

### Pre-harvest heat stress affects leaf physiology postharvest

SPAD readings were used as a measure of chlorophyll content (Fig. 1A). Overall, there was a general decline in chlorophyll with time of storage, but there was no significant effect of the pre-harvest heat treatment. Photosynthetic efficiency was significantly lower in heat-stressed leaves after 21 d of cold storage (Fig. 1B) compared with at harvest. There was no significant difference between control and stressed leaves at each storage time point, although *F*_v_/*F*_m_ was slightly (though not significantly) higher in heat-stressed leaves at harvest and lower in heat-stressed leaves compared with controls after 21 d of storage. Conductivity as a measure of ion leakage also showed a marked change after 21 d of storage (Fig. 1C), with a dramatic increase in the heat-stressed leaves compared with controls. The mean values were also slightly (but not significantly) higher in the heat-stressed leaves compared with controls at each time point and there was a slight upward trend with storage time. Stomatal aperture (measured as width/length from light microscopy images) increased slightly (but not significantly) with the heat treatment at harvest but then fell during storage, with a lot of variability (Fig. 1D). Stomatal conductance was lower in the heat-treated leaves at harvest, perhaps due to the hot humid environment, and fell with storage in both control and heat-treated leaves after 7 d (though not significantly) (Fig. 1E) Overall, pre-harvest heat stress affected physiological responses most after prolonged storage.

**Fig. 1.**
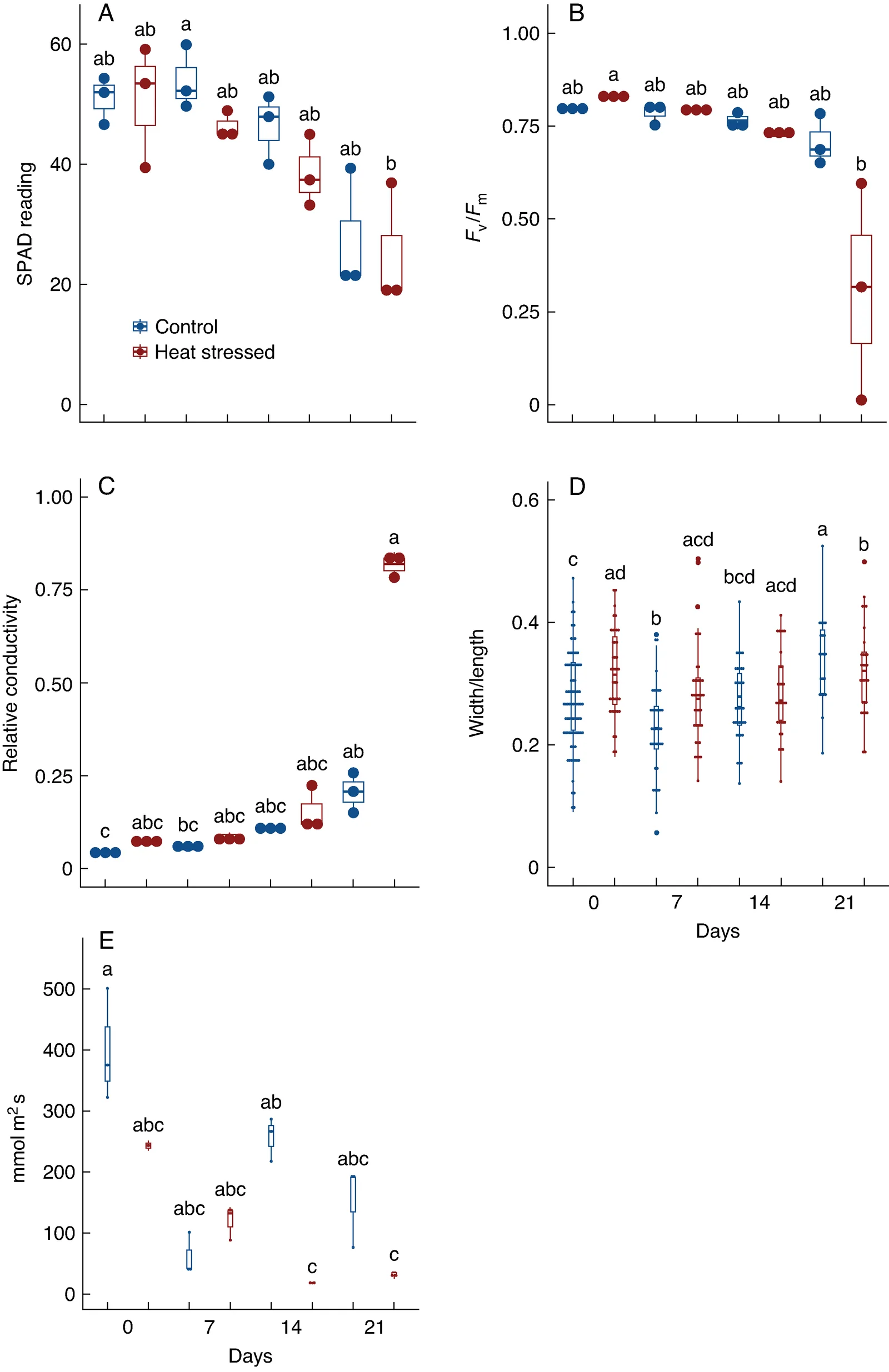
Physiological changes elicited by pre-harvest heat stress compared with a control at harvest (day 0) and during storage at 6 °C. (A) Chlorophyll content (B) Photosynthetic efficiency. (C) Ion leakage. (D) Stomatal opening. (E) Stomatal conductance. Data are mean ± standard deviation. (A–C) *n* = 3, (D) *n* > 20. Different letters indicate significant differences based on a Kruskal–Wallis test.

### Changes in VOC profiles precede physiological changes

As metabolic changes may be expected to precede physiological changes, VOC profiles were used as an initial proxy for assessing metabolic change in response to the same pre-harvest heat stress and 7 d of storage at 6 °C. Given the inherent variability of VOC responses, the experiment was repeated twice independently. Overall, 135 compounds were detected across the profiles of the four treatments, with 72 % (97 compounds) shared between the two repetitions of the experiment (Supplementary Data Table S3). The VOC family with the highest relative abundance was alkanes, followed by esters, aldehydes and alcohols (Table 1).

**Table 1.** Relative abundance of VOC families across the two repetitions of the experiment.

VOC family	Control at harvest	Stressed at harvest	Control postharvest	Stressed postharvest
Repetition 1				
Alcohols	30.23 ± 1.75a	32.87 ± 4.47a	22.3 ± 1.37b*	28.98 ± 1.23a*
Aldehydes	15.49 ± 1.91a*	8.94 ± 0.79a*	12.97 ± 4.12a	12.66 ± 2.03a
Alkadienes	0.28 ± 0.10a	0.19 ± 0.02a	0.22 ± 0.03a	0.22 ± 0.06a
Alkanes	2.89 ± 0.44a*	2.75 ± 0.44a*	4.10 ± 0.65b*	3.86 ± 0.17ab*
Alkenes	0.16 ± 0.04a*	0.17 ± 0.04a	0.12 ± 0.02a*	0.14 ± 0.02a
Alkynes	0.02 ± 0.01ab	0.01 ± 0.00b	0.03 ± 0.01ab	0.05 ± 0.03a
Amides	0.40 ± 0.12a	0.23 ± 0.04a	0.22 ± 0.03a*	0.24 ± 0.01a
Aromatics	1.14 ± 0.17a	1.07 ± 0.17a	0.91 ± 0.12a*	0.91 ± 0.11a
Carboxylic acids	0.05 ± 0.01a	0.02 ± 0.00a	0.04 ± 0.01a*	0.04 ± 0.01a
Esters	37.58 ± 3.62ab*	42.75 ± 5.46ab	48.24 ± 6.37b*	37.01 ± 0.43a
Ethers	0.00 ± 0.00a*	0.00 ± 0.00a*	0.00 ± 0.00a*	0.00 ± 0.00a*
Furans	0.26 ± 0.03ab	0.19 ± 0.04b	0.29 ± 0.09ab*	0.40 ± 0.07a*
Isothiocyanates	1.11 ± 0.21b*	2.01 ± 0.74ab	1.50 ± 0.27ab	3.13 ± 0.36a*
Ketones	7.00 ± 0.68a*	5.77 ± 0.89a*	5.23 ± 0.64a*	6.89 ± 0.40a*
Lactones	0.99 ± 0.56ab	0.21 ± 0.19b*	1.45 ± 0.26ab	2.44 ± 1.00a*
Nitroalkanes	0.10 ± 0.01a*	0.10 ± 0.02a	0.11 ± 0.03a*	0.12 ± 0.03a*
Sulphur compounds	0.52 ± 0.11a	0.85 ± 0.30a	0.56 ± 0.25a	1.47 ± 0.47a
Terpenes	0.26 ± 0.05ab*	0.34 ± 0.34b*	0.20 ± 0.01ab	0.16 ± 0.01a
Repetition 2				
Alcohols	28.24 ± 1.92a	26.47 ± 2.39a	27.26 ± 2.43a	25.58 ± 1.75a
Aldehydes	9.14 ± 1.32b	17.58 ± 0.88a	17.88 ± 3.98a	16.20 ± 2.87a
Alkadienes	0.18 ± 0.02a	0.21 ± 0.12a	0.17 ± 0.06a	0.19 ± 0.06a
Alkanes	1.29 ± 0.13ab	0.90 ± 0.08b	1.50 ± 0.36a	1.23 ± 0.11ab
Alkenes	0.29 ± 0.04ab	0.12 ± 0.03c	0.37 ± 0.11a	0.20 ± 0.08bc
Alkynes	0.01 ± 0.01a	0.01 ± 0.00a	0.11 ± 0.08ab	0.13 ± 0.06b
Amides	0.20 ± 0.09ab	0.29 ± 0.15b	0.10 ± 0.03a	0.19 ± 0.05ab
Aromatics	2.13 ± 0.95a	0.89 ± 0.09a	1.70 ± 0.36a	1.17 ± 0.53a
Carboxylic acids	0.05 ± 0.03ab	0.01 ± 0.00b	0.10 ± 0.03a	0.23 ± 0.25a
Esters	45.63 ± 3.59a	47.19 ± 4.22a	29.81 ± 3.41b	32.24 ± 6.17ab
Ethers	0.06 ± 0.01ab	0.03 ± 0.00b	0.16 ± 0.03a	0.16 ± 0.04a
Furans	0.21 ± 0.05a	0.21 ± 0.09a	0.70 ± 0.12b	0.57 ± 0.06ab
Isothiocyanates	0.62 ± 0.26ab	0.56 ± 0.17b	1.12 ± 0.19a	1.01 ± 0.17ab
Ketones	2.66 ± 1.11a	2.79 ± 0.87a	2.39 ± 0.59a	3.34 ± 0.93a
Lactones	0.77 ± 0.57a	0.87 ± 0.39ab	8.21 ± 4.46ab	8.52 ± 3.75b
Nitroalkanes	0.05 ± 0.01a	0.06 ± 0.01a	0.32 ± 0.10b	0.29 ± 0.05b
Sulphur compounds	0.34 ± 0.10a	0.29 ± 0.12a	0.84 ± 0.18b	0.61 ± 0.15ab
Terpenes	0.15 ± 0.03a	0.17 ± 0.02a	0.22 ± 0.05a	0.17 ± 0.01a

*Significant difference in abundance of the VOC family across the two repetitions of the experiment (*P* < 0/05).

Different letters indicate significant differences in relative abundance of the VOC family across treatments within each experiment repetition (*P* < 0.05).

Based on relative abundance (MetaboLights study identifier MTBLS12935), there was shift in overall VOC profile in response to both the pre-harvest stress and the postharvest storage in both repetitions of the experiment, although the classification error based on Random Forest analysis was higher for the effect of the stress treatment than the postharvest storage (Supplementary Data Fig. S1). Considering all samples together, discrimination was better in the second experiment (Fig. 2A, B), seen in less classification error and in more discrete grouping in the ordination plots. The combination of pre-harvest stress and postharvest storage (SP) created the most distinct profile, which was discriminated from all other treatments in both repetitions of the experiments with no classification error. When the stressed and control samples were compared separately at each time point there was complete discrimination at harvest and after 7 d of storage (Fig. 2C, F), while at intermediate time points of 2 and 5 d there was less complete discrimination (Fig. 2D, E).

**Fig. 2.**
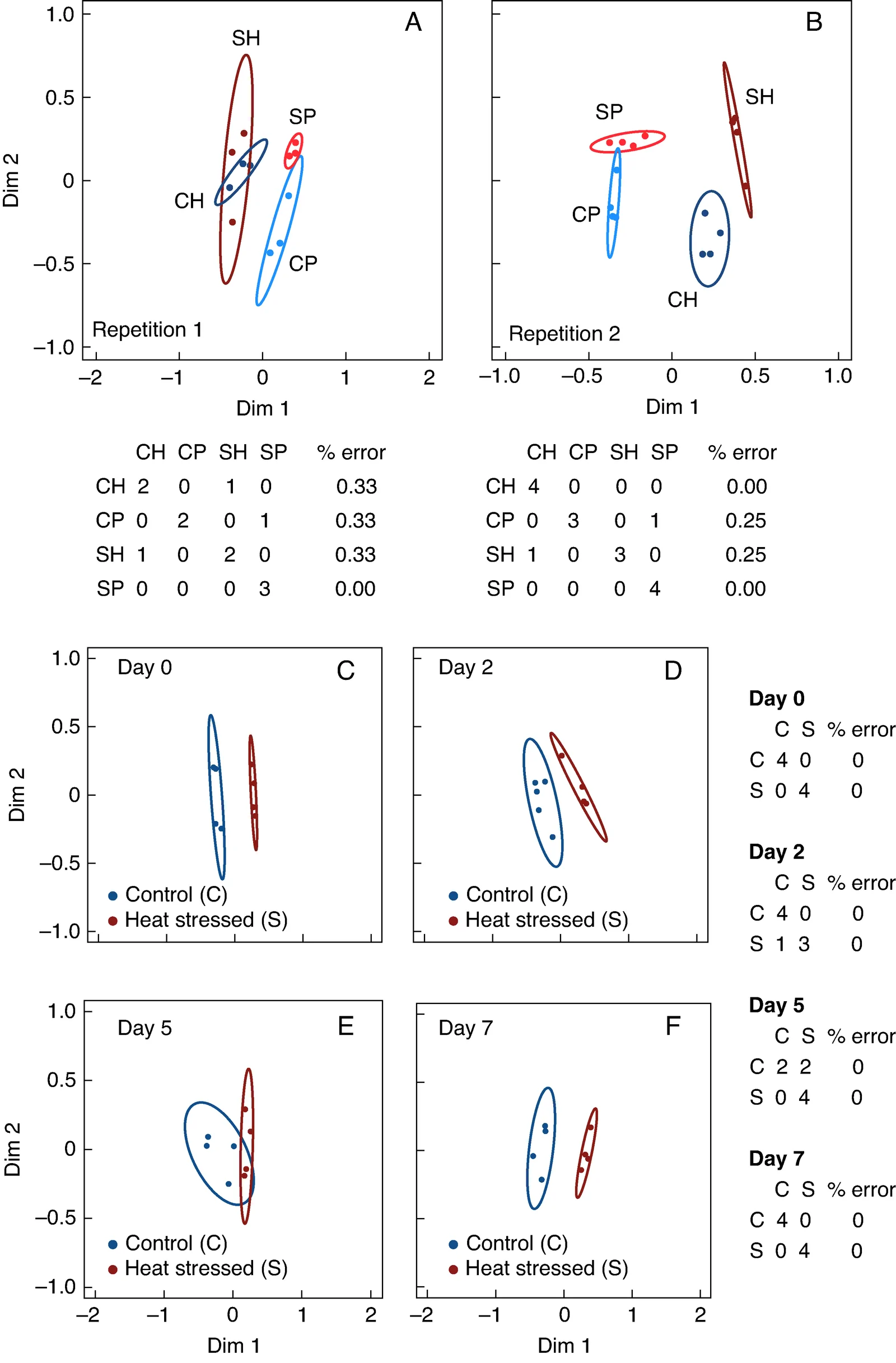
Effects on whole VOC profiles elicited by pre-harvest heat stress and storage at 6 °C. Ordination plots of Random Forest analysis (A) and (B) independent repetitions; leaves assessed at harvest (H) and after 7 d of storage (P), (C–F) separation by treatment: control (C) and stressed (S) on each day of storage. With each ordination plot is the percentage classification error. Ellipses show the 95 % confidence interval; *n* = 3 for (A) and 4 for (B–F).

The 97 compounds shared between both experiments were tested for their ability to discriminate the treatments. There was perfect discrimination between VOCs at harvest and after 7 d of storage (Fig. 3A), and good discrimination between stressed and control profiles (Fig. 3B). However, discrimination across the four samples was not as clear-cut compared with the whole profile of each experiment treated separately (Fig. 3C compared with Fig. 2A, B). Although many of the VOCs changed in abundance in response to the treatments (Supplementary Data Table S3). restricting the analysis to the eight most discriminatory compounds from the Random Forest analysis improved discrimination (Fig. 3 D, E). These eight VOCs included two compounds linked to green leaf volatiles (2-hexenal and hexen-1-ol). 2-Hexenal increased in relative abundance in response to the heat stress both before and after the chilled storage, although the effect was stronger (and statistically significant, *P* < 0.05) after storage. Storage *per se* had little effect on its relative abundance either in control or stressed samples (Fig. 3F). In contrast, (E)-3 hexanol changed in relative abundance primarily in response to storage but this was only a significant change (*P* < 0.05) in the heat-stressed leaves.

**Fig. 3.**
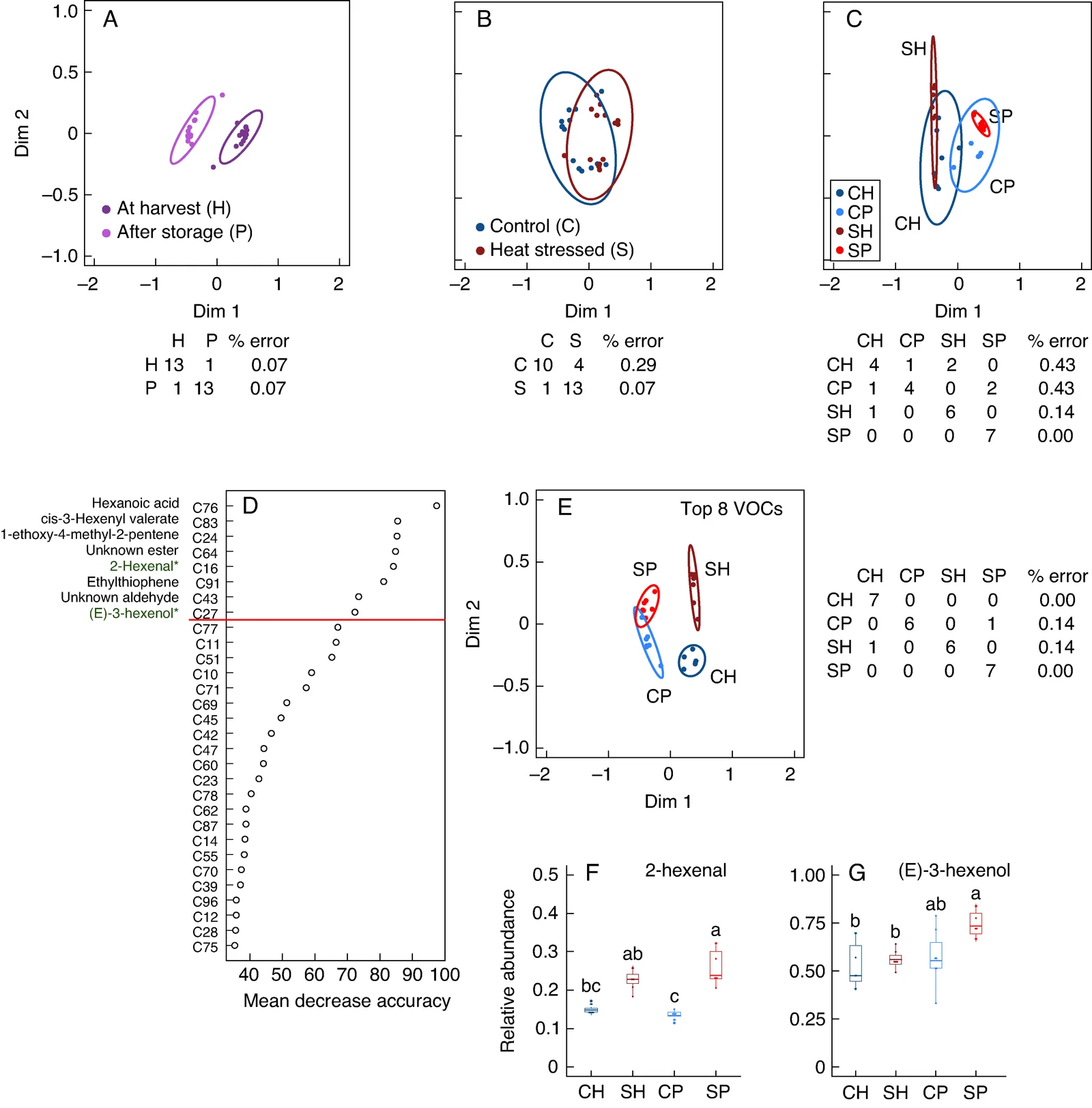
Effects on shared VOC profile across experiments elicited by pre-harvest heat stress (S compared with control C), and postharvest storage at 6 °C (P, compared with at harvest, H). Ordination plots of Random Forest analysis, separation by: (A) storage (B) stress treatment, (C) treatment and storage. (D) Mean decrease accuracy showing the most informative compounds (* indicates green leaf volatiles). (E) Ordination plot based on the top eight informative VOCs. (F) and (G) Relative abundance of the two green leaf volatile VOCs from (D). Alongside each ordination plot is the percentage classification error. Ellipses show the 95 % confidence interval; *n* = 14 for (A) and (B) and *n* = 7 for (C) and (E). Letters in (F) and (G) indicate significant differences based on a Kruskal–Wallis test (*n* = 7, *P* < 0.05).

### Metabolic changes are elicited by pre-harvest stress and storage

Since a shift was seen in the VOC profiles, the effect on the whole metabolome was tested. The whole metabolome profiles (7166 compounds; MetaboLights study identifier MTBLS12935) showed clear differences between harvest and storage time points, based on Random Forest analysis, and between control and heat-stressed samples (Fig. 4A, B) with completely correct classification. Indeed, all four samples were fully discriminated from each other (Fig. 4C). Due to the methodology used and the lack of complete databases, the majority of the compounds remained unidentified, but it was possible to assign a broad function to four out of the five most discriminatory compounds indicated through the Random Forest analysis (Fig. 4D). All four were lipids: two are tentatively annotated as glycerophosphoinositols and one as a sphingolipid, although the methodology does not allow firm identification. (Figure 4D). The relative abundance of these four lipids retained discrimination across the four samples (Fig. 4E) and showed good discrimination also for both treatment (Fig. 4F) and storage time (Fig. 4G). All four lipids increased in relative abundance in response to the heat stress at harvest (although this change was not always statistically significant) and p791.50616 (glycerophosphoinositol) abundance increased significantly (*P* < 0.05) in response to the stress even after postharvest storage. Abundance of all four compounds decreased in response to storage, although again in a few cases the change was not supported statistically (Fig. 4H–K).

**Fig. 4.**
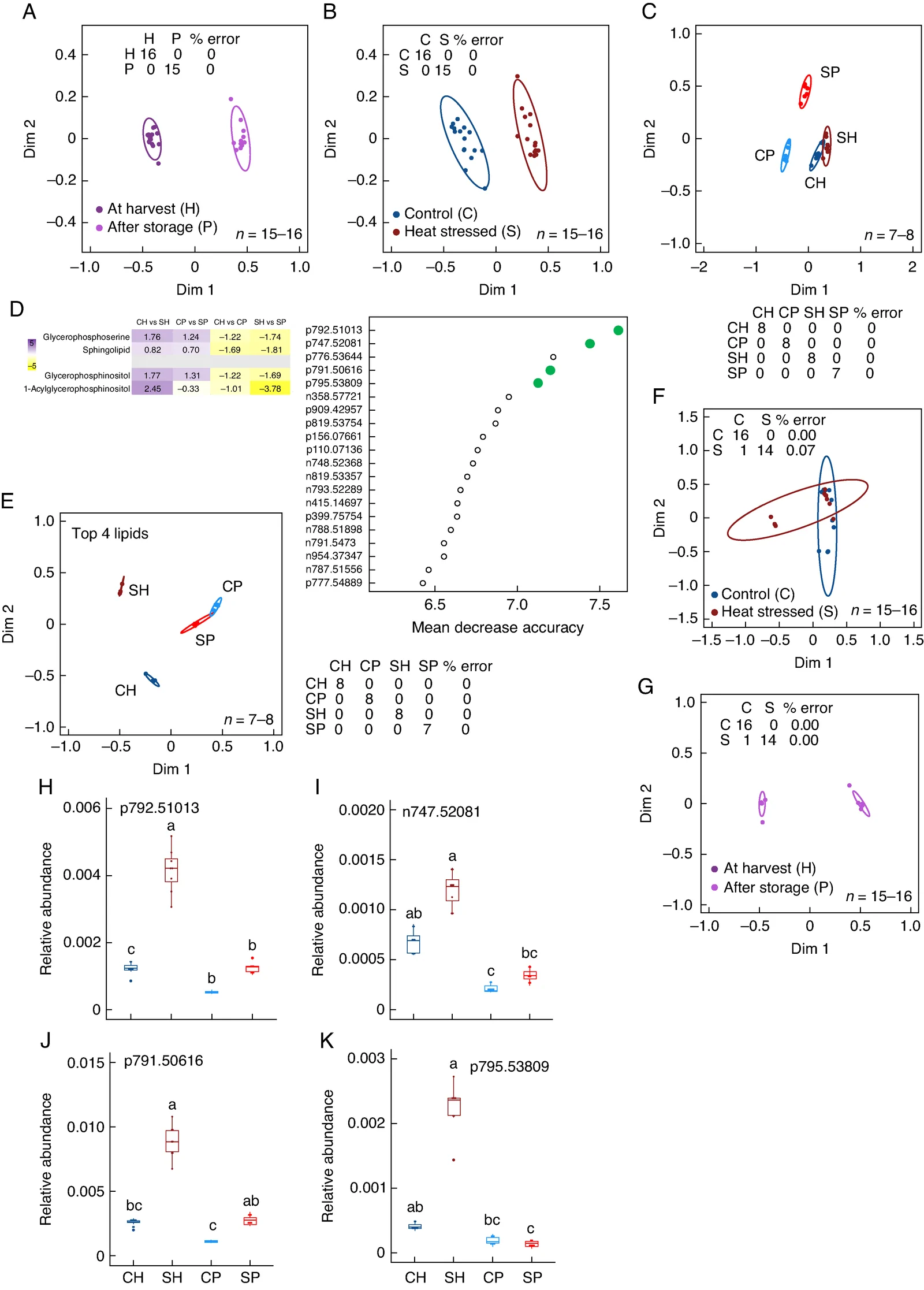
Effects on metabolome (FIE-HRMS) profiles elicited by pre-harvest heat stress (S and control, C and harvest, H), and storage at 6 °C (P). Ordination plots of Random Forest analysis comparing (A, G) days of storage, (B, F) treatment and (C, E) all samples based on all metabolites (A–C) and top four lipids (E–G) from mean decrease accuracy plot (green points). (D) Heat map of relative abundance of the four lipids in the different samples. Below/inside the ordination plots is the percentage classification error. Ellipses show the 95 % confidence interval; Letters in (H–K) denote significant differences based on a Kruskal–Wallis test (*P* < 0.05, *n* = 8).

Using GC–MS, it was also possible to assess the effects of the treatments on the relative abundance of selected sugars and amino acids (Supplementary Data Fig. S2). The relative abundances of all the sugars and amino acids analysed were highest in the leaves subjected both to the stress and the postharvest storage (SP) compared with all other treatments. Sucrose was also relatively high in the control samples both at harvest and after storage, but lowest in stressed leaves at harvest. Fructose was lowest at harvest in both treatments and then rose as a result of storage. Glucose remained fairly constant in relative abundance, while galactose was lowest in control leaves after postharvest storage. Most amino acids followed a similar pattern of abundance, being higher in the stressed leaves at harvest compared with the control (although for serine and proline there was no significant difference) and remaining fairly constant in the control in response to storage. However, all five amino acids were higher in relative abundance in the heat-stressed leaves after storage compared with the control after storage. Moreover, both serine and proline rose significantly in the stress-treated sample between harvest and 7 d of storage.

### Gene expression is affected by both pre-harvest stress and storage

Given the shifts in metabolism, and the stresses imposed, the effect on the expression of two previously studied stress-responsive genes (Spadafora *et al.*, 2019) was tested to assess if the pre- and postharvest stresses also affected gene expression. Both *DtNAC19* and *DtNAC59* were strongly upregulated in the stressed leaves as a result of postharvest storage, while the rise in expression as a result of storage was less marked in the control leaves (Supplementary Data Fig. S3). However, while at harvest the stress treatment slightly (though not significantly) repressed expression of both genes, after storage the stress slightly decreased expression of *DtNAC019* but increased expression of *DtNAC059.*

To assess gene expression changes more broadly transcriptomes of the four treatments were compared. A global transcriptome comprising 55539 contigs/transcripts ranging from 190 to 16 500 bp was generated from *de novo* assembly of RNA-Seq data produced from rocket leaves exposed to three days of heat pre-harvest, and controls at harvest (CH and SH respectively) and after seven days of storage under cold dark conditions (SP and CP respectively) (Supplementary Data Table S4). Of these, 38 406 contigs (69 %) could be matched to an *A. thaliana* gene using BLASTX. More differentially expressed genes (DEGs; cut-off of 1.4 < log2 FC < −1.4 and FDR/*P*_ad_j < 0.001) were seen as a result of storage (8203 and 6644, respectively, for stressed and control treatments) than as a result of the pre-harvest stress treatment. The latter resulted in more DEGs compared with the unstressed control following storage (952) compared with at harvest (438) (Supplementary Data Fig. S4). More DEGs were downregulated compared with upregulated in the comparisons (Fig. 5A, B) and most shared DEGs were between the CH vs CP and SH vs SP comparisons. Very few DEGs were shared between the CH vs. SH and CP vs SP comparisons. This indicates that although the effect of the pre-harvest stress was still detected after the storage period, a different subset of genes changed in expression. The largest group of DEGs uniquely affected by the treatments were those in SH vs SP comparison (1329 up- and 1585 downregulated) indicating a specific effect of the combined pre- and postharvest stress treatment.

**Fig. 5.**
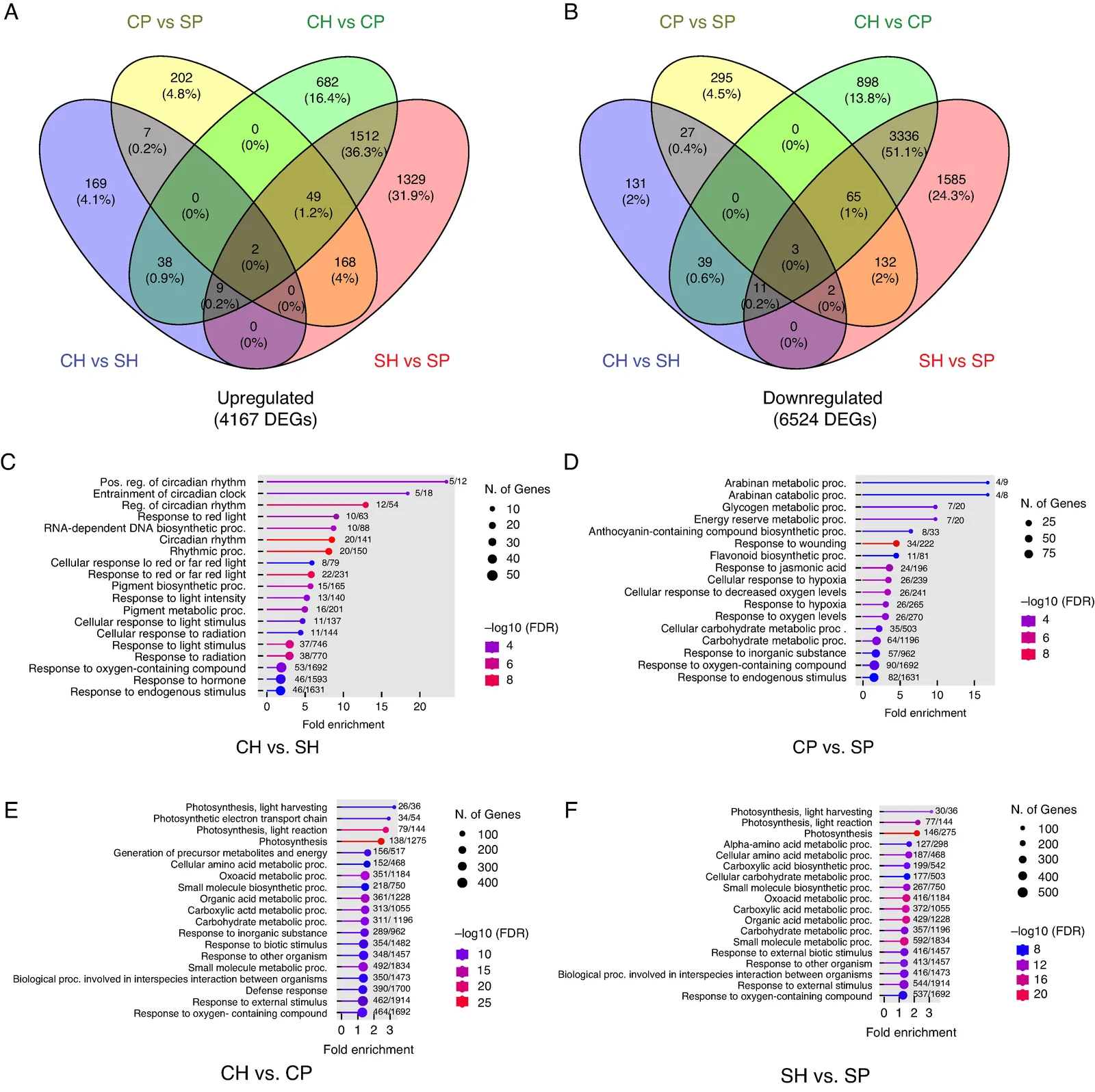
Transcriptome overview. Venn diagrams showing all (A) upregulated and (B) downregulated DEGs (log2 fold change >1.4; *P*adj < 0.001). C, control; S, heat-stressed; H, at harvest; P, after 7 d at 6 °C. Diagrams created using Venny (Oliveros, 2007). (C–F) most enriched GO terms (number of DEGs compared with all genes in that pathway found in the transcriptomes that showed significant alignment to *A. thaliana* genes. Created using ShinyGO v0.77 (Ge *et al.*, 2020).

Gene Ontology analysis was used to identify the terms most enriched by the treatments (Fig. 5C–F). Pre-harvest heat stress (CH vs SH) most significantly affected circadian rhythm associated genes, but these genes appeared less affected by the other treatments. In contrast, after postharvest storage the effect of pre-harvest heat stress was seen as a change in metabolic processes. Photosynthesis as well as a number of metabolic processes were affected by storage both in control (CH vs CP) and stressed (SH vs SP) leaves. Both at harvest and after storage some phytohormone changes were detected.

### Expression of phytohormone signalling genes and transcription factors is affected by both pre-harvest stress and storage

To validate the transcriptome data and explore the effects on phytohormone signalling in more detail, real-time qPCR was used to assess changes in expression of seven phytohormone-related genes (Fig. 6A–G) and compared with the transcriptome data. In general, the real-time qPCR confirmed the transcriptome data with the same direction of change in all cases, although changes were not always significant (*P* < 0.05) based on the real-time qPCR results.

**Fig. 6.**
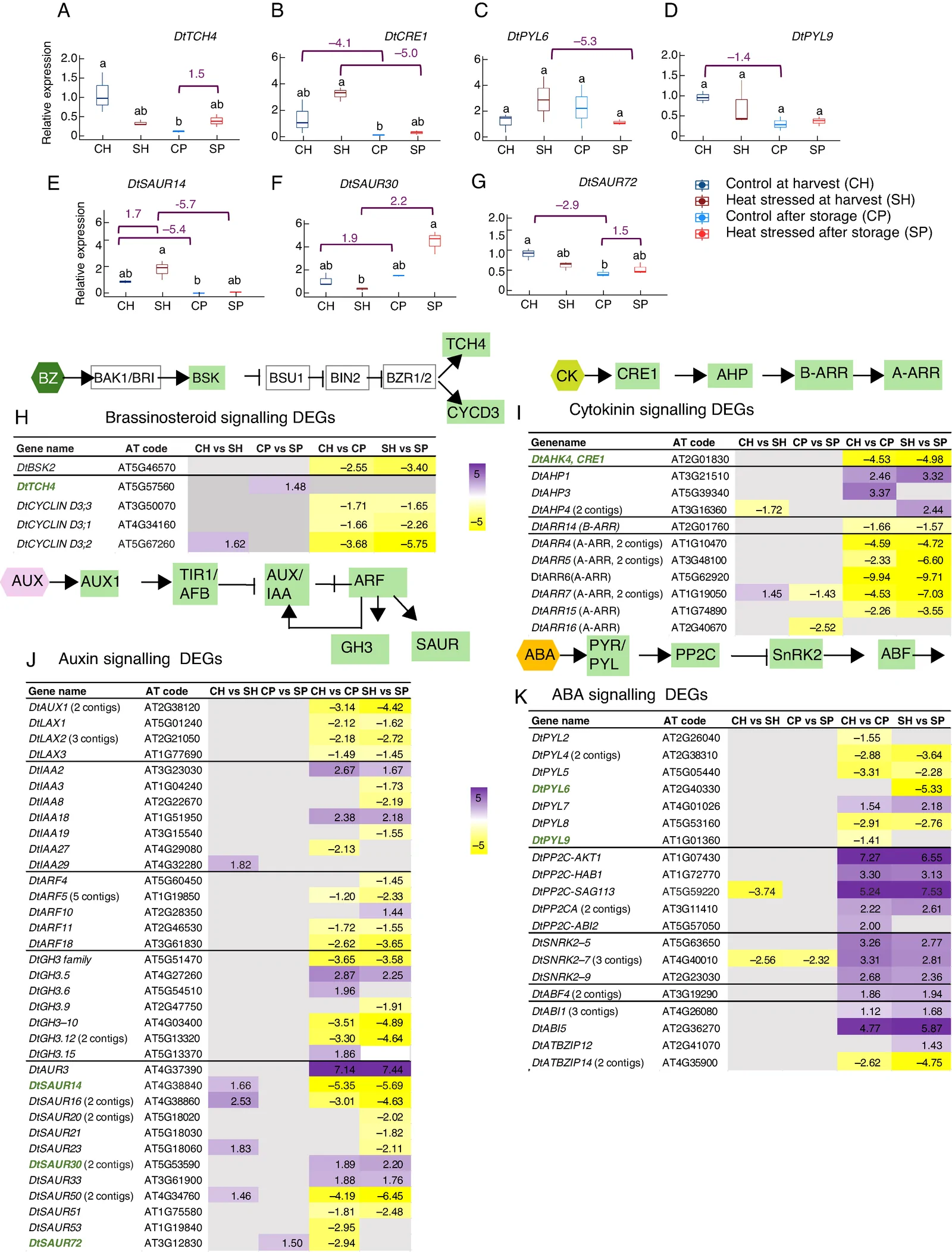
Effect on phytohormone-related genes elicited by pre-harvest stress (S) and controls (C) at harvest (H) and after 7 d of storage at 6 °C (P). (A) *DtTCH4*, (B) *DtCRE1*, (C) *DtPYL6*, (D) *DtPYL9*, (E) *DtSAUR1*, (F) *DtSAUR68*, (G) *DtSAUR98*. Mean ± standard deviation, *n* = 3; different letters indicate significant differences based on a Kruskal–Wallis test from real-time qPCR; log2 fold changes from the transcriptome data are shown in purple for each gene. (H–K) Phytohormone pathways (green shading indicates representation in the transcriptome DEGs) and heat maps of log2 fold change of genes with *Arabidopsis* codes and gene names; average is shown where there is more than one contig.

DEGs related to brassinosteroid signalling were upregulated by the pre-harvest stress treatment but downregulated by postharvest storage (Fig. 6H). *TCH4*, which in *Arabidopsis* responds to brassinosteroids (Iliev *et al.*, 2002), was upregulated by the heat treatment but only after postharvest storage. *DtBSK2* was downregulated in response to storage in both control and heat-stressed leaves, as were several cyclin D genes. The cytokinin receptor gene *DtCRE1* and several *A-RR* genes as well as one *B-ARR* gene were also downregulated by storage in both leaf treatment groups while AHP genes were mostly upregulated (Fig. 6I).

*DtAUX1* and three *LAX* genes were downregulated by storage in both control and stressed leaves, as were most *ARF* genes. *GH3* and *SAUR* genes showed a mixed response, with several *SAUR* genes upregulated by the heat stress at harvest but not after storage, and one upregulated after storage but not at harvest (Fig. 6J). The response of ABA signalling genes was consistent with their role in the pathway. Thus, PYL genes were mostly downregulated by storage while PP2C SNRK and ABF genes were upregulated (Fig. 6K).

A total of 232 contigs were functionally annotated as transcription factors (TFs), matching 198 different *Arabidopsis* transcription factors and falling into 40 different families (Supplementary Data Fig. S5) Most TF DEGs (160) changed in expression in response to stress plus storage (SH vs SP), with slightly fewer changing in the control (CH vs CP comparison; 132). Heat stress at harvest and after storage (CH vs SH and CP vs SP) elicited 16 and 21 different TF DEGs respectively. Out of these, 11/16 and 12/21 DEGs were upregulated by the stress treatment before and after storage respectively. Four TF genes changed in expression in relation to heat stress either before or after storage; three of these were upregulated. Of the 132 TF genes affected by storage in the control leaves (CH vs CP), there more were downregulated (76) than upregulated (56), and this pattern was the same in the DEGs for storage in the stressed leaves (SH vs SP): 91 downregulated compared with 69 upregulated.

The ERF TF family was represented by the largest number of different members (22) followed by bHLH (16), MYB-related and MYB (15 and 11 respectively), and NAC represented by 12 different members of the family. In some TF families, e.g. AP2, B3, bHLH, GATA, HD-ZIP and TCP, most of the DEGs were downregulated in response to cold storage in either control or stressed leaves or both. In contrast, in other families, such as bZIP, C2H2, Dof, G2-like, HSF, NAC, NF-Ya, NF-YB, NF-YC and WRKY, most family members were upregulated in response to cold storage in one or both leaf treatment groups.

### Effect of pre-harvest heat stress and cold storage on cell death and senescence-related genes

Given the rise in ion leakage after 21 d of storage (Fig. 1), changes in the expression of genes related to programmed cell death (PCD) and senescence were examined to assess if this cellular response detected after 21 d of storage might be already detected as gene expression changes after 7 d of storage (Supplementary Data Fig. S6). Genes both positively and negatively correlated with PCD onset or progression were both up- and downregulated in response to storage in both stressed and control leaves, with very little change in response to stress at harvest (Supplementary Data Fig. S6A, B). Most of the changes were similar after storage between the stressed and control leaves.

In relation to senescence, of perhaps greatest significance is the strong upregulation of *DtSAG12* after storage in both control and stressed leaves as this is a widely accepted marker specific to plant organ senescence (Lohman *et al.*, 1994, Supplementary Data Fig. S6C). Indeed, most of the genes positively associated with leaf senescence were upregulated after the dark, cold storage, although a few were downregulated and some genes associated negatively with senescence were upregulated (Supplementary Data Fig. S6C, D).

### Green leaf volatile genes and VOCs, and jasmonic acid biosynthesis-related genes are affected by both stress and storage

As noted above, two GLV VOCs and two other VOCs derived from the LOX pathway increased in relative abundance in response to postharvest storage (Fig. 3). The transcriptome was mined for genes related to the LOX pathway, finding three LOX genes, one hydroperoxide lyase and one aldehyde reductase (Fig. 7). All three genes were downregulated in response to the postharvest storage both in stressed and control leaves, and the *DtLOX3* gene was also downregulated in response to the stress treatment but only after storage.

**Fig. 7.**
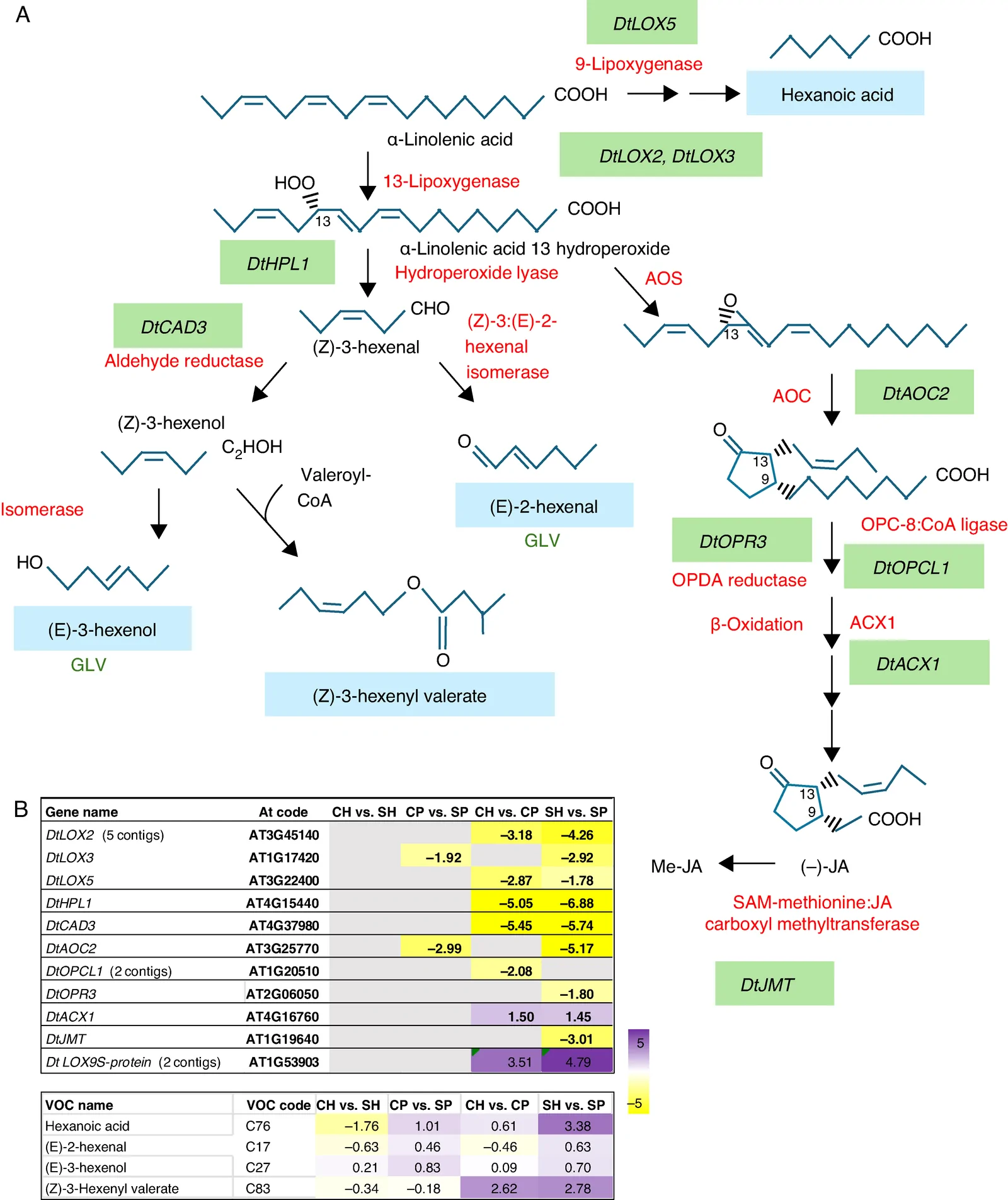
Green leaf volatile (GLV) and JA biosynthesis pathways elicited by pre-harvest stress (S) and controls (C) at harvest (H) and after 7 d of storage at 6 °C (P). (A) Compounds are in black and enzymes are in red font. Most discriminatory VOCs (GLVs indicated) are in blue shading. Shaded green are DEGs encoding the relevant enzymes. (B) Heat maps of log_2_ fold change for each DEG; average is shown where there is more than one contig. Closest match *Arabidopsis* codes are shown for each contig and LOX pathway-derived VOCs.

Genes related to JA biosynthesis were also found in the transcriptomes, with *DtAOC2*, *DtOPR3*, *DtJMT* and *DtOPCL1* downregulated while *DtACX1* was upregulated in response to storage (Fig. 7). Five JAZ-encoding genes were also DEGs, mostly downregulated by the storage, but DtJAZ8 was upregulated by stress treatment though only after storage (Supplementary Data Fig. S7). Several JA-responsive genes were downregulated, but *DtMAPKKK14* and *DtPDF1.2A* were upregulated in response to storage.

### DEGs include several genes encoding enzymes involved in sphingolipid biosynthesis

Based on the changes in lipid abundance detected via the metabolomic analysis (Fig. 4), expression of related genes was assessed in the transcriptomes. Ten DEGs related to sphingosine biosynthesis were present in the transcriptomes (Fig. 8). Most were upregulated by the storage treatment in both stressed and control leaves, in contrast to compound n747.52081, identified as discriminatory from the analysis of the metabolome (Fig. 4) which increased in relative abundance in response to the heat stress both before and after storage but was downregulated in both leaf samples after storage.

**Fig. 8.**
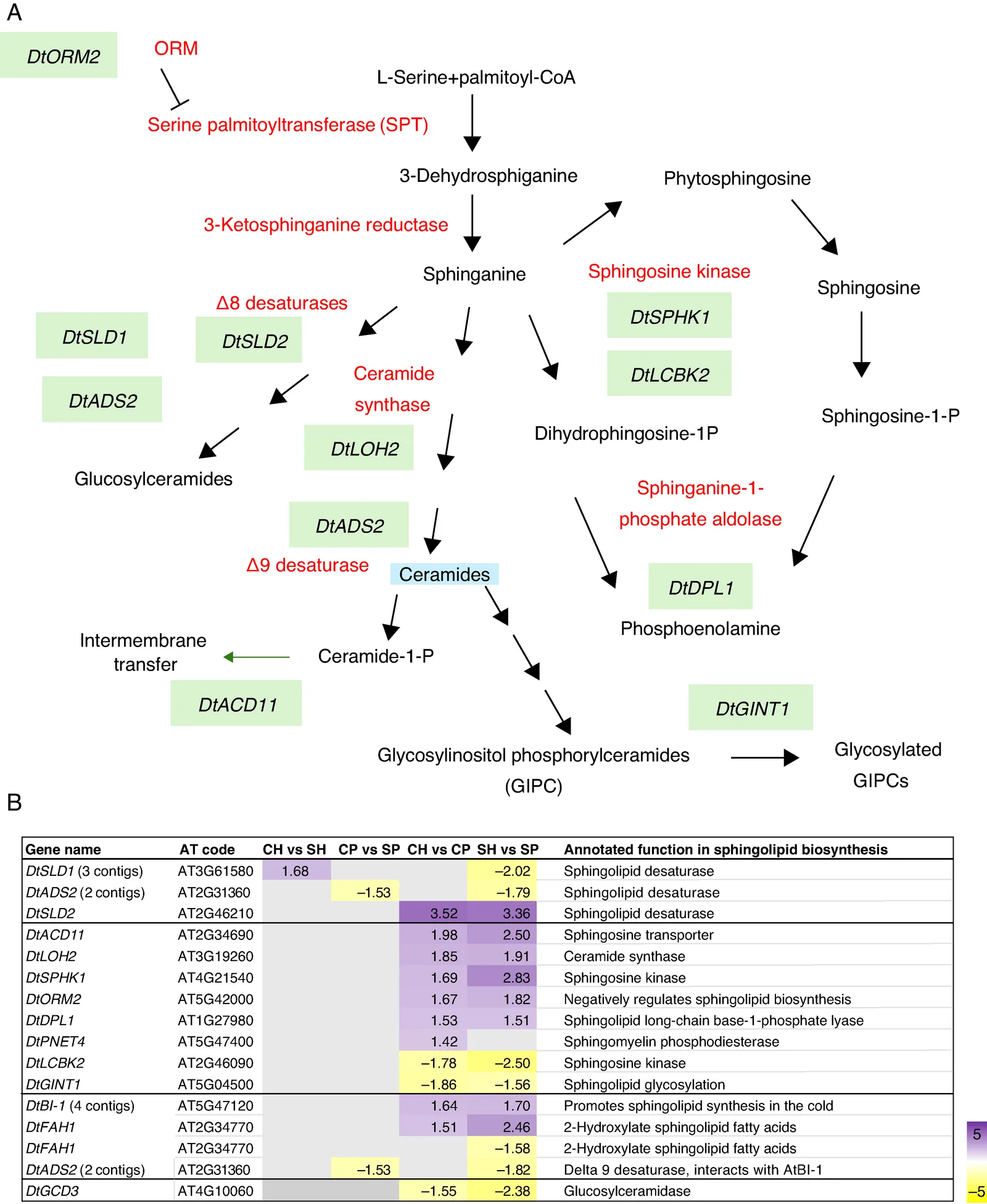
Sphingolipid pathways. (A) Compounds are in black and enzymes are in red font. Most discriminatory compound classes are in blue shading. Shaded green are DEGs encoding the relevant enzymes. Direction of relative abundance or log_2_ fold change in relation to storage. (B) Log_2_ fold change for each DEG; average is shown where there is more than one contig. Closest match *Arabidopsis* codes and gene names are shown for each contig.

## DISCUSSION

Exposure of rocket salad to heat stress during growth affected gene expression, volatilome, metabolome and physiology of the leaves after postharvest storage. Effects on the transcriptome, volatilome and metabolome were evident after 7 d of cold storage while physiology was more affected after longer storage periods.

### The treatment elicited mild but not severe heat stress

The heat stress imposed was above the optimal temperature range for rocket salad (Jasper *et al.*, 2020), although only 5 °C higher than the maximal optimum. This is hot for the UK but not as severe as might be experienced by rocket salad in Southern Europe, where commercial cultivation methods can expose the plants to temperatures of over 40 °C (Cacini *et al.*, 2024). The physiological data indicate that the leaves in this experiment have not been exposed to severe stress. Under severe stress a lowering of *F*_v_/*F*_m_ and SPAD readings immediately after the stress treatment would be expected, due to damage to the photosynthetic apparatus (Zahra *et al.*, 2023). Cell death would also increase just after severe heat stress (Distéfano *et al.*, 2017), which would have been seen as a rise in conductivity, and this was not seen here. In addition, severe heat stress would activate *Hsf* and *Hsp* gene expression (Ohama *et al.*, 2017), which was not detected in the transcriptomic analysis at harvest. Responses to mild heat stress are coordinated by PIF4/PIF5 (Casal and Balasubramanian, 2019) with an upregulation of their transcripts (Stavang *et al.*, 2009). Here there was an upregulation of *DtPIF5* by heat stress at harvest, while after 7 d of chilled storage *DtPIF4* was upregulated, again suggesting that the treatment resulted in mild heat stress.

### Markers of senescence indicate an early phase even after 7 d of storage

Although the mild pre-harvest heat stress resulted in physiological changes after prolonged cold storage, at harvest heat-stressed leaves showed few signs of stress; indeed, *F*_v_/*F*_m_ was slightly (though not significantly) elevated in the heat-treated leaves. The lack of significant change in either chlorophyll or photosynthetic activity after 7 d of dark cold storage in either stressed or control genes contrasts with some previous studies (Cavaiuolo *et al.*, 2015; Spadafora *et al.*, 2019) but suggests that here the leaves have not yet entered physiological senescence. However, these data contrast with the increase seen here in *DtSAG12* expression, an accepted marker of leaf senescence, as well as upregulation of *DtNAC089* associated with ER-stress induced PCD (Yang *et al.*, 2014), and *SAG21* associated with ROS induction (Mowla *et al.*, 2006). Thus, it seems that after 7 d of cold storage, before photosynthesis is affected, senescence is being activated at a transcriptional level before cellular and physiological changes are detectable.

### Both the mild heat stress and postharvest storage elicited changes in the circadian clock

In *Arabidopsis*, *PIF4*/*5* interact with circadian clock components. Several other clock components were upregulated as a result of heat stress at harvest. In *Arabidopsis PIF4* is downregulated by ELF3, ELF4 and LUX at dusk (Herrero *et al.*, 2012; Fig. 9). However, leaves here were collected mid-morning so *DtPIF4* expression is predicted to still be high, and was upregulated in the heat-stressed leaves, in agreement with data from *Arabidopsis* (McClung and Davis, 2010). After a much shorter period of heat stress (1 h at 37 °C), *CCA1*, *PRR7* and *PRR9* expression was upregulated in *Arabidopsis* (Blair *et al.*, 2019) but *LHY* was reduced. Here orthologues of all four rocket genes were upregulated following the stress treatment; however, heat exposure was longer here, which may have affected *DtLHY* expression. The effect of mild heat stress on these genes after 7 d of cold/dark treatment was very different. *DtPIF5* expression was unaffected, but *DtPIF4* expression was upregulated, and expression of other components of the clock – *DtELF4*, *DtPRR7* and *DtPRR5* – were downregulated. Circadian rhythms are disrupted postharvest when leaves are maintained in constant darkness (Goodspeed *et al.*, 2013). The change in expression of these key clock regulators is consistent with a disruption of the clock in rocket salad leaves both by heat stress and later by cold/dark storage.

**Fig. 9.**
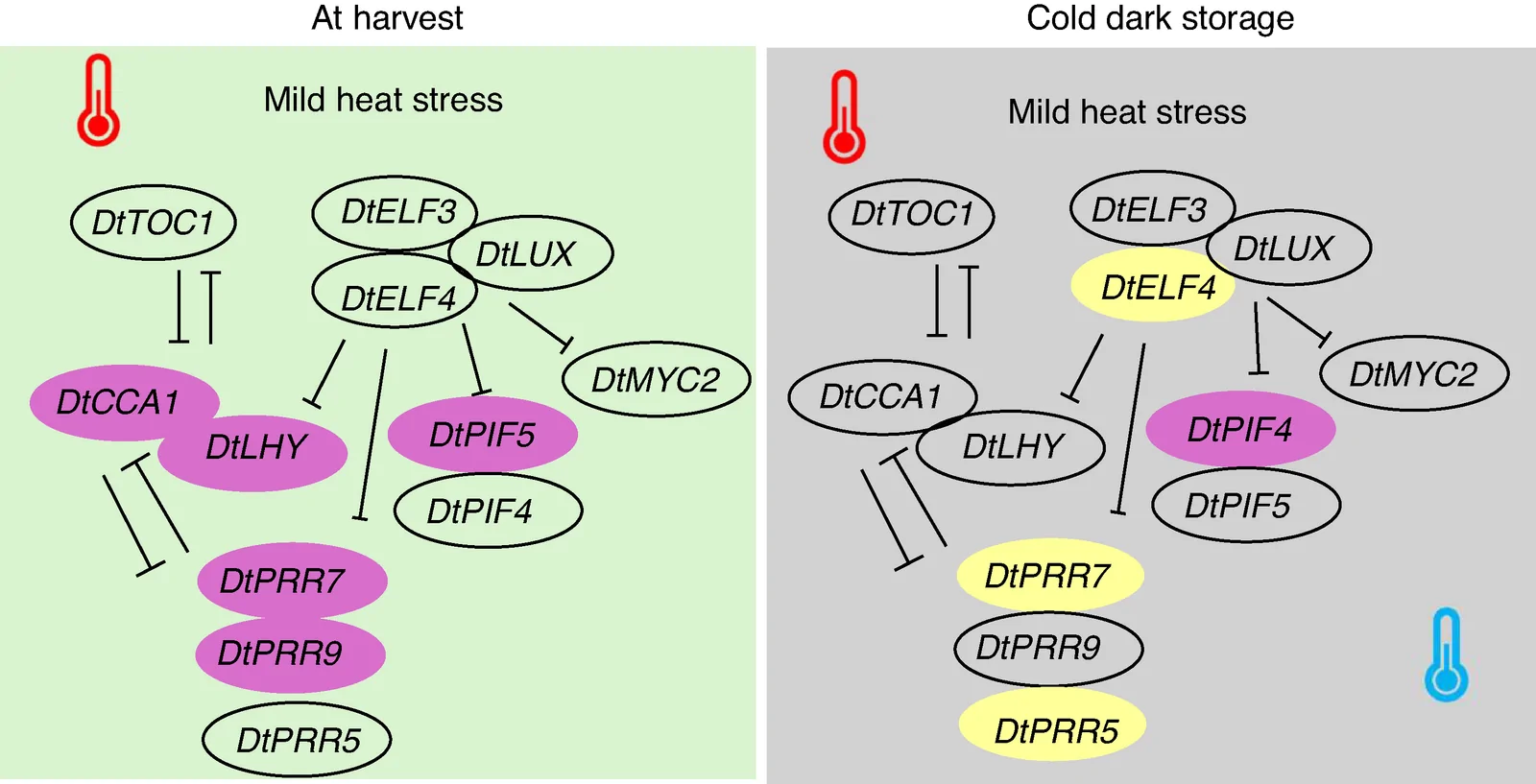
Circadian clock genes at harvest and after 7 d of cold dark storage. Purple shows upregulation and yellow shows downregulation of *D. tenuifolia* gene expression in response to heat stress.

### Gene expression changes elicited by the postharvest storage fit a model based on *Arabidopsis* responses to dark and cold

*PIF4*/*5* are also involved in promoting senescence in response to dark through the activation of ethylene and ABA signalling pathways. *ABI5*, which in *Arabidopsis* is downstream of *PIF4/5*, was strongly upregulated by the cold/dark storage of the rocket leaves. Expression of several *DtPP2C* and *DtSNRK* genes involved in ABA signalling was also affected by dark storage. An ACS gene (most homologous to *AtACS8*) involved in ethylene biosynthesis was also upregulated. Other downstream players of *PIF4/5*: *DtANAC092* and *DtANAC002, SGR1* and *NYC1* were also upregulated as well as most of the SNAC-As (Takasaki *et al.*, 2015). These data are all in accordance with the model developed in *Arabidopsis* for dark-induced senescence (Liebsch and Keech, 2016; Fig. 10). *ANAC019* and *AtSAG113* are also direct targets of *PIF4* and *PIF5* in *Arabidopsis* (Li *et al.*, 2021). Here neither *DtNAC019* nor *DtSAG113* was upregulated DEGs in relation to heat stress at harvest or after storage; in fact, *DtSAG113* was downregulated by heat stress at harvest. However, both genes were strongly upregulated by storage treatment, in line with the upregulation of *DtPIF4/5* in response to dark/cold postharvest stress.

**Fig. 10.**
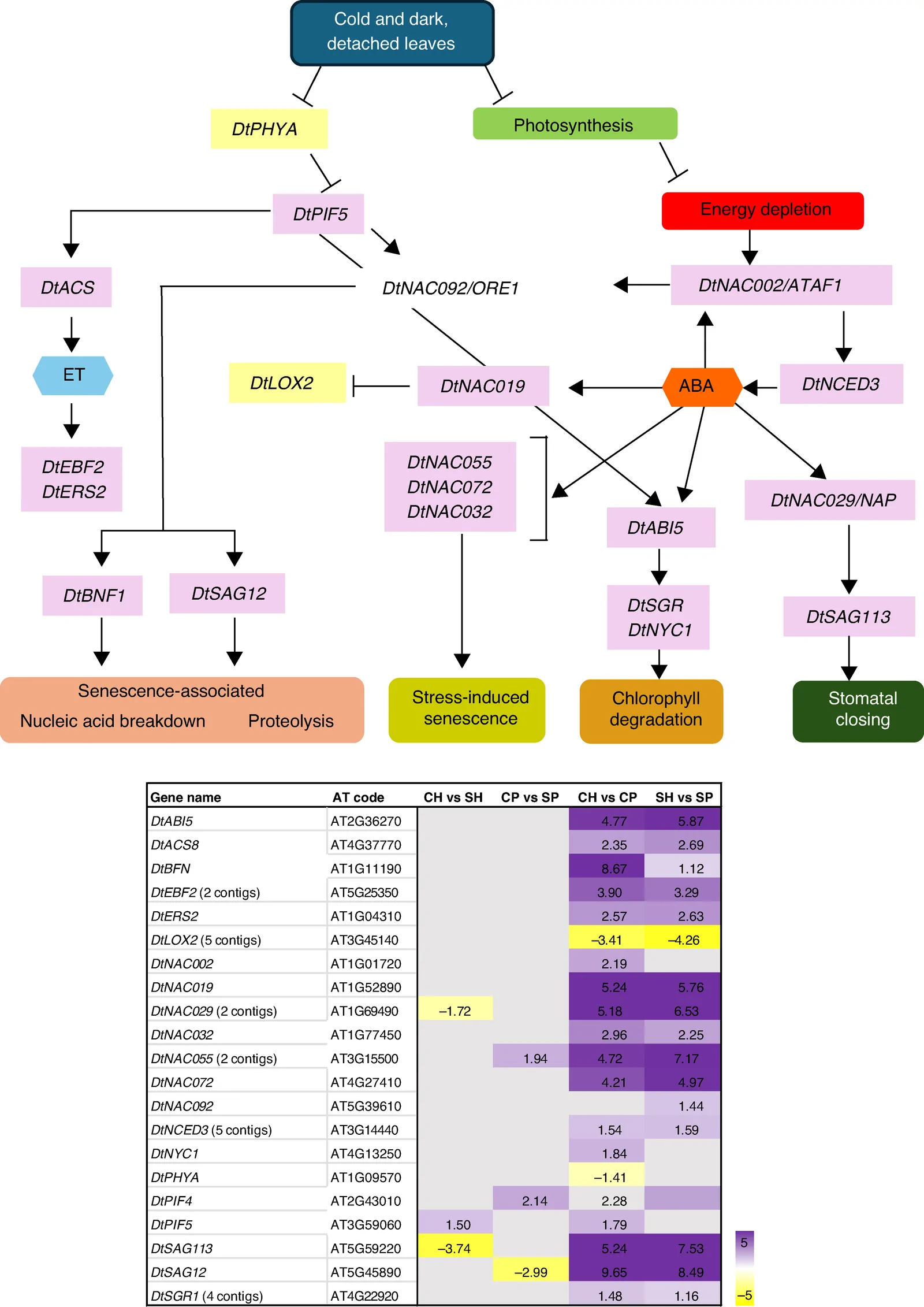
Model of gene network activated by cold and dark stress in control leaves and resulting in stomatal closure, and stress-induced senescence characterized by a reduction in photosynthetic efficiency and chlorophyll degradation. Model based on data from *Arabidopsis*. Purple shows upregulation and yellow shows downregulation of *D. tenuifolia* gene expression after 7 d of post-harvest storage at 6 °C.

### Multiple stresses perturb expression of phytohormone-related genes consistent with effects of cold and dark on *Arabidopsis*

Expression of other phytohormone signalling genes was also affected by heat stress. *DtIAA29*, involved in auxin signalling, was upregulated in response to heat stress at harvest, in line with Li *et al.* (2021). In *Arabidopsis* this gene is also directly regulated by PIF4/PIF5 and hence agrees with the *DtPIF4*/*5* upregulation seen here. Other auxin signalling genes were upregulated by the stress treatment. In seedlings elevated temperature promotes auxin biosynthesis and hypocotyl growth. Here *DtYUC8*, involved in auxin biosynthesis and another direct target of PIF4 (Sun *et al.*, 2012), was upregulated by heat stress at harvest. This indicates a similar signalling and growth response to that seen in *Arabidopsis*. After storage, auxin signalling genes were generally downregulated; this is again in line with a negative effect of the dark and cold stresses on growth, tipping the leaves instead towards senescence. Brassinosteroids act together with auxin in promoting hypocotyl elongation in *Arabidopsis* (Stavang *et al.*, 2009). However, here the only brassinosteroid-related genes upregulated in response to heat stress were brassinosteroid targets, *DtTCH4* and *DtCYCLIN D3;2*. These genes are related to growth rather than specifically to brassinosteroid signalling (Dewitte *et al.*, 2007; Shinohara *et al.*, 2017). In *Arabidopsis* seedlings brassinosteroid gene expression was activated at later stages of growth compared with auxin-related genes in response to growth at elevated temperature (Stavang *et al.*, 2009); thus the 3-day exposure in more mature leaves may not have been sufficient to upregulate this temperature response. Whereas ABA signalling genes were upregulated in response to the cold dark stress, auxin and brassinosteroid DEGs were downregulated, as were genes related to cytokinin signalling, reflecting the inhibitory effect of postharvest conditions on leaf expansion.

### Combined cold and dark storage conditions trigger both DREB-dependent and independent gene expression changes

As well as dark stress, postharvest storage also exposed the leaves to cold stress. Given the upregulation during the postharvest storage of *DtRVE3* and *DtRVE5* (members of the RVE8 family), *DtZAT12* and *DtLHY*, this might suggest the activation of a DREB1-independent signalling pathway (Kidokoro *et al.*, 2022). However, *COR27* and *COR28*, which are also involved in this pathway, were not activated by cold storage in rocket salad. This is probably due to the dark conditions as both genes are light-induced and downregulated in the dark (Kahle *et al.*, 2020). There is also evidence from the transcriptome that the DREB signalling pathway is involved, since *DtDERB1A* was activated both by stress at harvest and in the control by postharvest storage. In contrast, *DtDREB1D* expression was activated only by postharvest storage in the heat-stressed leaves.

*LNK1* in *Arabidopsis* is regulated by *RVE8* (Sorkin *et al.*, 2023) and upregulates COR genes in response to cold. *DtLNK1* was upregulated by stress at harvest but postharvest was only upregulated in the control leaves. *REV4*/*8* also upregulate *ERF53* and *ERF54* in *Arabidopsis* in response to heat shock (Li *et al.*, 2019); however, *DtERF54* was downregulated by postharvest storage both in heat-stressed and control seedlings. This may be because the *RVE4*/*8*-dependent activation of *ERF54* in *Arabidopsis* is very rapid and in response to a more severe heat shock. *Arabidopsis ERF54* is upregulated by dark treatment (Xu *et al.*, 2010) as well as cold (Kilian *et al.*, 2007) and promotes senescence. The downregulation of *DtERF54* may be due to the stress combination, or a different function of this gene in *D. tenuifolia*.

### Persistent effects of heat stress after cold/dark storage may be amplified by green leaf volatiles

As well as affecting the leaves transcriptionally, the pre-harvest and postharvest stresses also altered the VOC profile. The greater effect was elicited by postharvest storage, as might be anticipated from previous studies (Spadafora *et al.*, 2016, 2019). However, the pre-harvest stress effect persisted and was still detectable after 7 d of cold dark storage. This indicates that even this mild stress had significantly affected the metabolome. Two green leaf volatiles were amongst the VOCs whose relative abundance was most significant in the changes elicited by the combined pre- and postharvest stress. 2-Hexenal, in particular, was significantly more abundant in stressed than control leaves after postharvest storage. This VOC has been shown to elicit changes in expression of large numbers of genes also affected by heat stress (Yamauchi *et al.*, 2015). Nine of the top 100 genes reported by Yamauchi *et al.* (2015) as most upregulated by 2-hexenal treatment were also amongst the DEGs elicited by pre-harvest stress here and detected after 7 d of storage (Supplementary Data Fig. S8). One of these genes is *WRKY46*, which in *Arabidopsis* is required for 2-hexenal induced anti-herbivore responses (Hao *et al.*, 2024) and is also ABA-regulated. Three of the other nine genes are also ABA-responsive in *Arabidopsis*, and two are involved in priming plants for biotic and abiotic stresses. These genes may be of relevance in VOC signalling postharvest in response to the combined pre-harvest heat stress and postharvest combined dark, cold, biotic, dehydration and wounding stresses.

### Regulation of LOX-derived VOCs during stress treatments is likely post-translational

Seventeen contigs, representing 11 LOX pathway genes, were amongst the DEGs (Fig. 8). The downregulation of the majority of these DEGs both in response to the heat stress and the postharvest stress contrasts with the increase in relative abundance of the four VOCs deriving from the LOX pathway that were amongst the most discriminatory compounds for the treatments. This suggests that the enzymes for the synthesis of the VOCs may be already present at harvest and become more active with time. A mismatch between lipoxygenase activity and its gene expression postharvest has been previously reported in broccoli (Gomez-Lobato *et al.*, 2012). The two LOX-related DEGs that were upregulated with postharvest storage were *DtACX1* and a putative *DtLOX9S* gene. These are both likely to be involved in the branch of the pathway that leads to jasmonate biosynthesis, which fits with the upregulation of other JA-related DEGs, including two JA responsive genes (*DtJUL1* and *DtMAPKKK14*; Supplementary Data Fig. S7). This might be due to the initiation of senescence which is also associated with an increase in jasmonate biosynthesis and signalling in *Arabidopsis* (Balbi and Devoto, 2008). However, several jasmonate-related genes were downregulated with storage, especially after heat treatment. This is consistent with an important role for post-translational regulation of JA biosynthesis (Wasternack and Song, 2017). Unexpectedly, all five JAZ genes that changed in expression were downregulated by the cold/dark treatment. This is in contrast to a previous report (Yu *et al.*, 2016), where four of them (JAZ1, 2, 8 and 10) were upregulated in response to ambient dark treatment. Thus the cold may be repressing this activation.

### Lipid metabolism changes show opposite responses to heat and cold/dark stress, but related gene expression is most affected postharvest

Cold also affects lipid metabolism and hence composition (Huby *et al.*, 2020) and this was seen both as a change in the expression of genes related to lipid biosynthesis and in lipid metabolites. A number of genes related to sphingolipid biosynthesis were upregulated by the cold/dark treatment in both stressed and control leaves, including *DtBI-1*, *DtFAH1*, orthologues of genes involved in ceramide modification (Nagano *et al.*, 2014). However, *DtSLD1* and *DtADS2* were downregulated in stressed leaves postharvest. Ceramidase genes putatively involved in sphingolipid breakdown (Li *et al.*, 2015) were either not amongst the DEGs or were downregulated (Fig. 9). However, the relative abundance of all four of the long-chain lipids, including a sphingolipid, that were amongst the five most discriminatory compounds in the metabolome were reduced in abundance postharvest. This discrepancy between metabolome results and gene expression may reflect the complexity of the metabolome or indicate post-translational regulation of this pathway.

### Conclusions

The exposure to a 3-d period of climate-relevant heat stress results in mild heat stress responses that are persistent through a realistic time frame of postharvest cold/dark storage in rocket salad. Postharvest cold/dark storage has the greater effect on gene expression and results in preparation for senescence initiation, involving chlorophyll and macromolecular degradation. The network of expression changes fits models developed from *Arabidopsis*, validating transfer of knowledge from the model to the crop. However, many *Arabidopsis* genes were represented by multiple rocket contigs, suggesting further complexity. A rise in (E)-2-hexenal is mirrored by a rise in eight genes previously identified in *Arabidopsis* as responding to this GLV. However, changes in other LOX-pathway VOCs and lipids contrast with gene expression and suggest post-translation control of these pathways postharvest. These responses to the multiple stresses eventually lead to physiological changes that would affect the quality of the salad.

## Supplementary Material

mcaf243_Supplementary_Data

## Data Availability

Sequencing data have been deposited in the NCBI Sequence Read Archive (http://www.ncbi.nlm.nih.gov/Traces/sra) under BioProject ID: ERP171275. VOC and metabolome data have been deposited in the MetaboLights repository (https://www.ebi.ac.uk/metabolights/study identifier MTBLS12935).

## SUPPLEMENTARY DATA

Supplementary data are available at *Annals of Botany* online and consist of the following. Table S1: list of contaminant VOCs excluded from the analysis. Table S2: list of all primers used. Table S3: list of VOCs. Table S4: RNAseq data. Figure S1: effects on whole VOC profiles elicited by pre-harvest heat stress and storage at 6 °C. Figure S2: relative contents of sugars and amino acids. Figure S3: real time qPCR of stress-responsive genes. Figure S4: MA plots of differentially expressed genes. Figure S5: effect of pre-and postharvest stresses on transcription factor gene expression. Figure S6: effect of pre-and postharvest stresses on cell death, senescence and autophagy-related gene expression. Figure S7: pathway and heat map of the DEGs related to jasmonic acid. Figure S8: DEGs whose expression changes in response to pre-and postharvest stresses and listed by Yamauchi *et al* (2015) as upregulated by 2-hexenal.
