# Supplementary material for: Pre-harvest heat stress affects rocket salad leaf transcription and metabolism at harvest and after chilled postharvest storage
Source: Ann Bot. 2025 Nov 13:mcaf243. Online ahead of print. doi: 10.1093/aob/mcaf243 (PMC13737583; doi:10.1093/aob/mcaf243)
Supplement: Supplementary file 1 [file mcaf243_Supplementary_Data.zip › Alotaibi et al Supp Figs 1-8 FINAL REVISED.pdf]

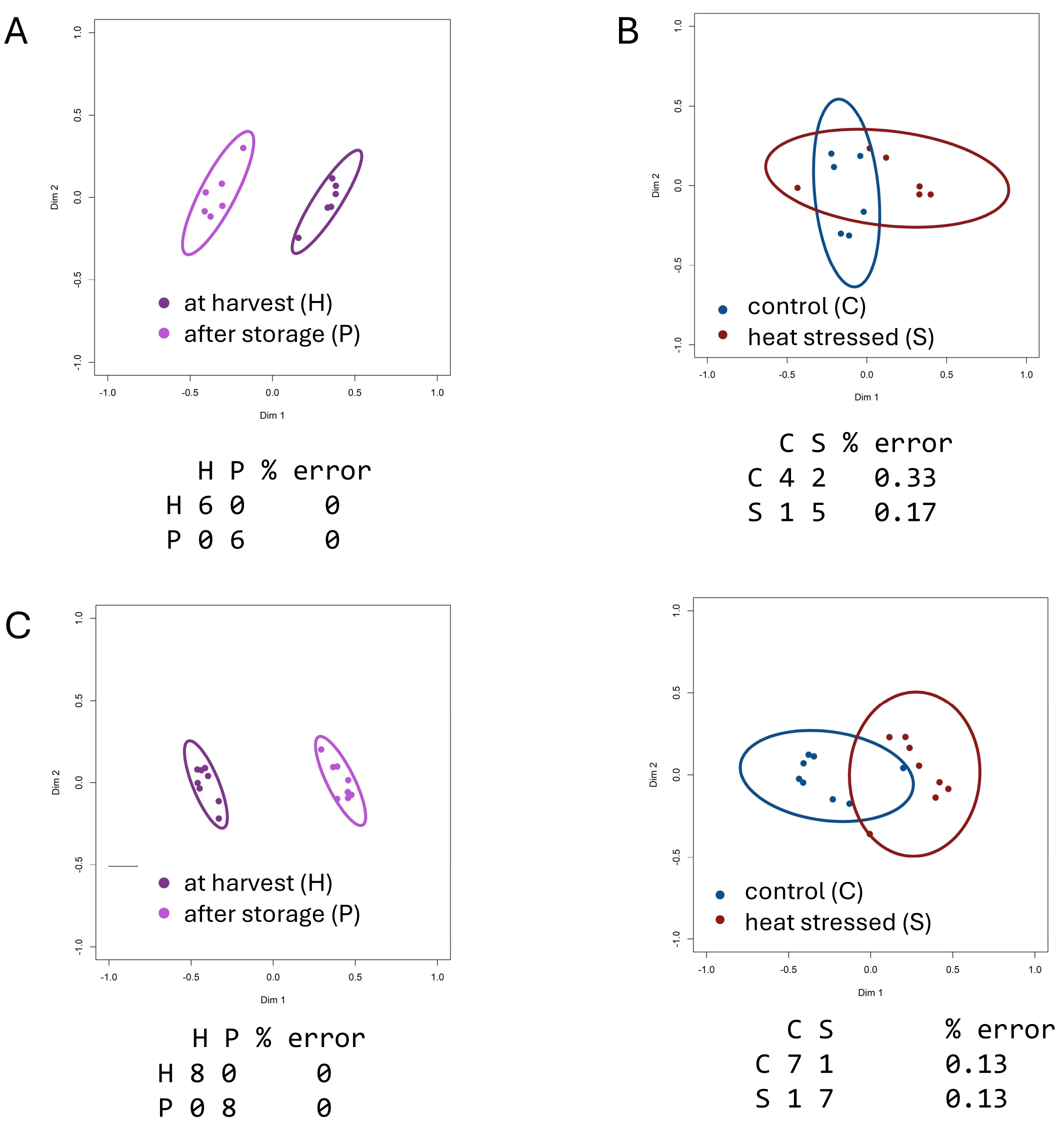

Supplementary Fig. 1 Effects on whole VOC profiles elicited by pre-harvest heat stress, and storage at 6 °C. Ordination plots of Random Forest analysis comparing (A and C) days of storage, (B and D) treatment in A and B, first experiment, C and D repeat experiment. Leaves assessed at harvest (H) and after 7 days of storage (P), following control (C) and stress (S) treatment. Below the ordination plot is the % classification error. Ellipses show the 95% confidence interval; n 15-15 for (A) and (B), and 7-8 for (C).

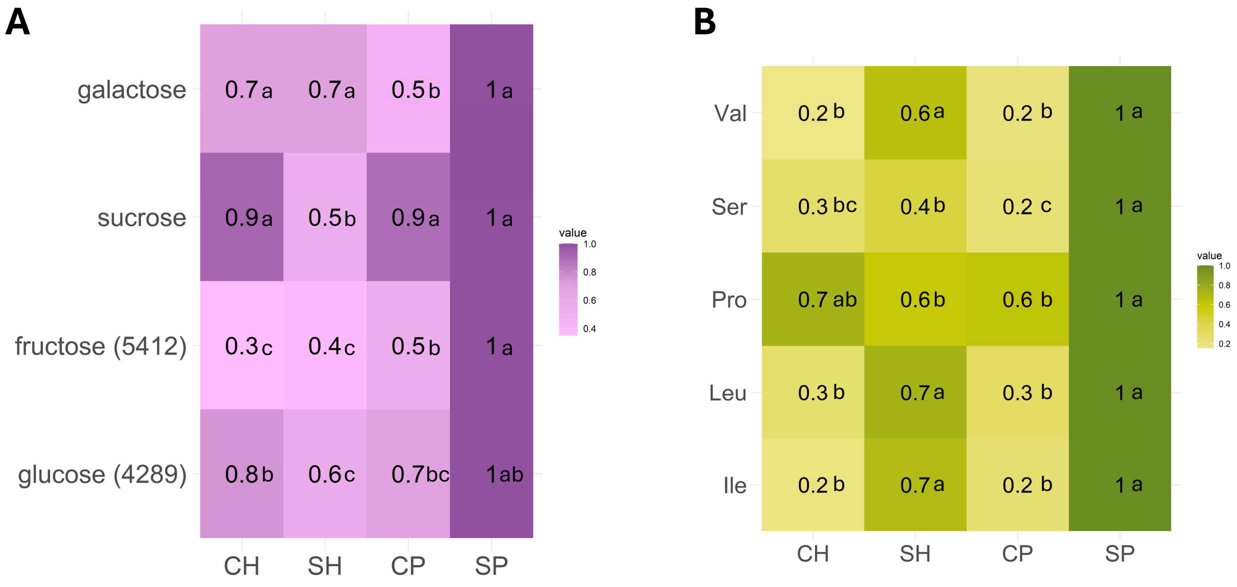

Supplementary Figure 2. Relative content (GC-MS) of (A) sugars, (B) amino acids, normalised to highest value. Letters indicate significant differences (p < 0.05, n=8)

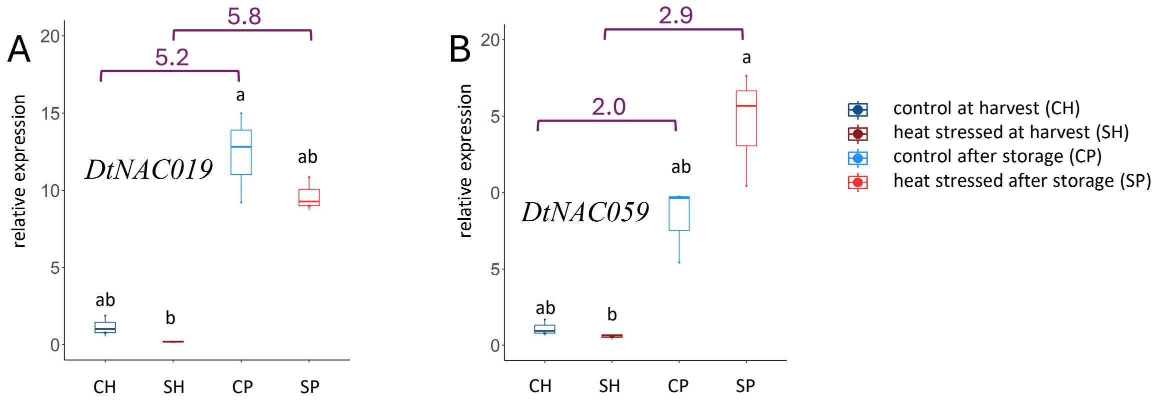

Supp Figure 3. Effect on stress-responsive genes elicited by pre-harvest stress and storage at 6 °C: (A) *DtNAC019* (B) *DtNAC059* mean ± SD, n = 3; different letters indicate significant differences (p<0.05) based on a Kruskall-Wallis test.

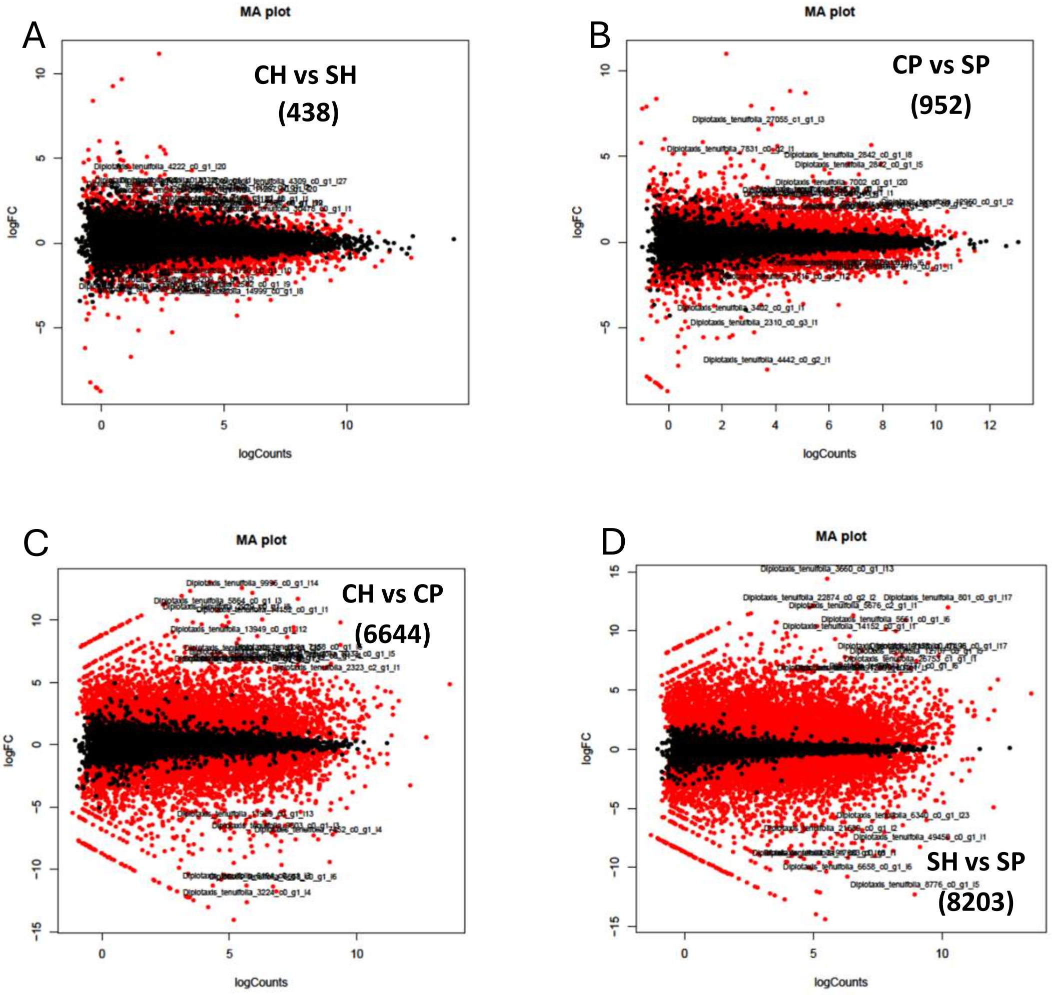

Supplementary Fig. 4. MA plots of differentially expressed genes identified the in four comparisons (black dots are gene expression > padj cut off of 0.001; red dots are DEGs, cut-off of 1.4 < log2 fold change < -1.4 and FDR/Padj < 0.001. (A) stressed and control leaves at harvest (CH vs. SH), (B) stressed and control leaves after 7 d of storage (CP vs. SP), (C) control at harvest and after storage (CH vs. CS), and (D) stressed leaves at harvest and after storage (SH vs. SP).In brackets the number of DEGs for each comparison.

Family	gene name	AT code	CH vs SH	CP vs SP	CH vs CP	SH vs SP
AP2	*DtANT*	AT4G37750			-2.35	-2.91
	DtPLT3	AT5G10510			-1.83	-3.10
	DtAIL5	AT5G57390			-1.44	-2.43
	DtTOE2 (2 contigs)	AT5G60120	1.44		-1.48	-2.98
ARF	*DtARF5*	AT1G19850			-1.71	-3.37
B3	*DtREM32* (2 contigs)	AT2G24700		1.84	-4.30	1.70
	DtHSI2	AT2G30470			2.05	
	DtREM1	AT3G19184				-1.90
	DtNGA4	AT4G01500		-1.64		
	DtREM1	AT4G31610			-2.12	-3.95
	DtDPA4 (2 contigs)	AT5G06250			-2.47	-4.05
	DtREM20 (3 contigs)	AT5G18000			-2.01	-2.24
BBR-BPC	*DtBPC5*	AT4G38910				1.48
BES1	*DtBEH4*	AT1G78700			-2.18	-2.44
bHLH	*DtPIF3*	AT1G09530			3.20	2.21
	DtPIF4	AT2G43010		2.14		2.28
	DtPIF5	AT3G59060	1.50		1.79	
	DtPIF7	AT5G61270		1.46	-2.59	
	DtPRE6	AT1G26945				-1.73
	DtBHLH96	AT1G72210			5.79	
	DtHBI1	AT2G18300		2.32	-5.06	-4.33
	DtBHLH091	AT2G31210			-1.98	
	DtBHLH130	AT2G42280			-2.51	-1.82
	DtbHLH70	AT2G46810			-4.93	-5.05
	DtBHLH 3	AT3G07340			-2.51	-2.47
	DtbHLH87 (2 contigs)	AT3G21330			-2.23	-3.09
	DtPRE5	AT3G28857				-1.98
	DtBHLH059	AT4G02590				1.77
	DtBIM1	AT5G08130				-2.22
	DtBNQ2	AT5G15160			-6.96	-6.93
	DtBHLH071	AT5G46690			-1.49	-2.12
	DtCIB2	AT5G48560			-1.48	-2.15
bZIP	*DtBZIP52* (2 contigs)	AT1G06850				-1.44
	DtABI5	AT2G36270			4.77	5.87
	DtBZIP5	AT3G49760				4.81
	DtGBF6	AT4G34590			2.25	1.91
	DtBZIP14	AT4G35900			-2.62	-4.75
	DtGBF1	AT4G36730				1.46
	DtBZIP63	AT5G28770	2.06		2.02	
	DtTGA1	AT5G65210				2.64
C2H2	*DtC2H2-like*	AT1G26610			-2.31	-1.97
	DtZAT10	AT1G27730				2.89
	DtIDD4	AT2G02080			1.56	1.54
	DtZAT4	AT2G45120			2.08	1.66
	DtIDD11	AT3G13810			1.45	
	DtZAT9	AT3G60580				-1.61
C3H	*DtFES1*	AT2G33835			2.65	3.78
	DtZNF1 (2 contigs)	AT3G02830			-2.21	-2.34
	DtC3H58	AT5G18550	-1.41		-2.36	-2.42
	DtTZF5	AT5G44260			4.06	3.90
CO-like	*DtCOL9*	AT3G07650				-1.49
	DtBBX8	AT5G48250		-1.57		-2.16
CPP	*DtSOL2*	AT4G14770			-2.19	-3.32
	DtTCX7	AT5G25790			2.07	0.34
DBB	*DtSTO*	AT1G06040	1.91			-2.54
	DtBBX22	AT1G78600	2.59			-2.78
Dof	*DtCDF6*	AT1G26790		-3.13	6.66	
	DtCDF5 (2 contigs)	AT1G69570		-2.04	2.83	1.58
	DtDOF2.1	AT2G28510				-3.16
	DtDOF2.4	AT2G37590			1.95	1.71
	DtDOF2	AT3G21270			2.04	
	DtCDF2	AT5G39660			2.73	2.44
	DtDOF5.3	AT5G60200			-1.63	-1.98
ERF	*DtAPD1*	AT4G13040			-1.62	-2.05
	DtCRF1	AT4G11140				-4.03
	DtCRF3	AT5G53290	1.78			-4.14
	Dt-DREB family A-5	AT4G31060			-1.69	
	DtDREB1A	AT4G25480	2.31		3.78	
	DtDREB1D	AT5G51990				3.76
	DtDREB26 (2 contigs)	AT1G21910			-5.19	-3.37
	DtDREB2B	AT3G11020			1.58	
	Dt-DREB-A-2	AT3G57600			-5.49	-4.20
	Dt-DREB-A-5	AT1G77640			-4.55	-4.29
	DtERF018	AT1G74930				2.09
	DtERF041 (2 contigs)	AT5G11590			-2.85	-2.20
	DtERF1	AT3G23240			3.63	4.15
	DtERF104	AT5G61600		1.97		
	DtERF106	AT5G07580				-2.58
	DtERF115	AT5G07310			6.75	5.09
	DtERF34	AT2G44940			-2.87	
	DtERF39	AT4G16750		-1.41		-1.74
	DtERF54	AT4G28140			-1.50	-2.12
	DtDEAR4 (2 contigs)	AT4G36900			1.81	2.87
	DtRAP2.3 (2 contigs)	AT3G16770			1.93	3.14
	DtRAP2.6L	AT5G13330			4.35	4.23
FAR1	*DtFRS1*	AT4G19990			-1.44	
G2-like	*Dt Homeodomain-like*	AT1G69580			1.48	2.64
	DtPHR1	AT4G28610	1.56			-1.78
	DtHHO5	AT4G37180	-1.98		1.73	3.93
	DtGLK2	AT5G44190	3.41		3.01	
GATA	*DtGATA11* (2 contigs)	AT1G08010		1.43	-1.51	-2.05
	DtGATA17	AT3G16870			-2.30	
	DtGATA8	AT3G54810			-3.21	-3.37
	DtGATA4 (2 contigs)	AT3G60530		-1.85	-2.05	-1.48
	DtGATA3	AT4G34680			1.84	2.34
	DtGATA19	AT4G36620			-2.80	-4.61
	DtGATA12	AT5G25830			-1.67	-1.92
	DtGATA5 (2 contigs)	AT5G66320				-2.00

Family	gene name	AT code	CH vs SH	CP vs SP	CH vs CP	SH vs SP
GEBP	*DtSTKR1*	AT4G00270			5.64	8.65
GRAS	*DtSCL28* (2 contigs)	AT1G63100			-4.13	-4.24
	Dt-GRAS family	AT3G50650			-1.55	-2.09
GRF	*DtGRF8*	AT4G24150			1.90	2.09
	DtGRF7	AT5G53660				-1.61
HB-other	*DtPDF2* (2 contigs)	AT4G04890				-1.48
HD-ZIP	*DtHDG12*	AT1G17920			-1.61	
	DtHB4	AT2G44910			-4.77	-5.37
	DtHB-1 (2 contigs)	AT3G01470			1.65	
	DtHAT1 (2 contigs)	AT4G17460			-3.72	-2.74
	DtATML1	AT4G21750			-1.96	-2.20
	DtHB53	AT5G66700				1.65
HSF	*DtHSFB4*	AT1G46264			-2.15	-1.99
	DtHSFB2B (2 contigs)	AT4G11660				2.53
	DtHSFA4A	AT4G18880				1.59
	Dt -winged helix TF	AT4G19630			2.07	1.99
	DtHSF4	AT4G36990			1.41	2.66
	DtHSFA3	AT5G03720			-1.90	-1.49
	DtHSFB2A	AT5G62020	-2.93		2.10	5.32
	DtHSFA7A	AT3G51910			8.38	7.07
LBD	*DtLBD4*	AT1G31320				-2.20
	DtAS2	AT1G65620			1.73	
	DtLBD11	AT2G28500			-3.18	-2.84
	DtASL29	AT3G47870			-3.64	-3.29
	DtLBD31	AT4G00210				2.39
	DtASL1	AT5G66870			3.45	
	DtASL39	AT5G67420		1.45		
MICK MADS	*DtAGL2*	AT5G15800			-4.52	
	DtAGL8	AT5G60910				1.71
M-type MADS	*DtAGL93* (2 contigs)	AT5G26950			4.66	4.42
	DtAGL84	AT5G49420				2.20
MYB	*DtMYB47*	AT1G18710			-2.61	
	DtMYB3 (2 contigs)	AT1G22640				2.36
	DtMYB95	AT1G74430			-3.82	-2.29
	DtMYB2 (2 contigs)	AT2G47190			4.88	5.15
	DtMYB12	AT2G47460			-2.97	-1.51
	DtMYB106	AT3G01140			-4.77	-5.47
	DtMYB108	AT3G06490			3.15	2.70
	DtMYB30	AT3G28910			-2.23	
	DtMYB77	AT3G50060		-1.49		-2.53
	DtMYB102	AT4G21440				2.36
	DtMYB111	AT5G49330			-2.40	-2.73
	DtMYB96	AT5G62470			1.67	1.93
	DtMYBS1 (2 contigs)	AT1G49010			-4.03	-4.49
	DtMYB4	AT4G38620				-2.60
	DtDIV2	AT5G04760				1.54
MYB-related	*DtLHY1* (2 contigs)	AT1G01060	2.80		4.71	
	DtRL5	AT1G19510				-2.08
	DtMYBD	AT1G70000				-1.44
	DtMYBL2	AT1G71030				-1.59
	Dt Homeodomain-like	AT1G74840			-1.50	
	DtRL6	AT1G75250	2.78		-5.39	-8.78
	DtRL4 (2 contigs)	AT2G18328			-7.43	-9.26
	DtRVE8	AT3G09600	2.23			
	DtRVE5	AT4G01280				1.53
	DtRVE1	AT5G17300			3.76	2.92
	Dt-MYB-family	AT5G56840			-5.97	-4.54
NAC	*DtNAC001*	AT1G01010				-2.49
	DtNAC002	AT1G01720			2.19	
	DtNAC019	AT1G52890			5.24	5.76
	DtNAC025	AT1G61110				1.84
	DtNAC029	AT1G69490	-1.72		5.23	6.78
	DtNAC032	AT1G77450			2.96	2.25
	DtNAC036 (3 contigs)	AT2G17040			-4.22	-2.77
	DtNAC047 (2 contigs)	AT3G04070			5.17	5.15
	DtNAC052	AT3G10490				1.45
	DtNAC055	AT3G15500		1.94	5.24	9.91
	DtNAC056	AT3G15510				1.44
	DtNAC060	AT3G44290			5.88	5.81
	DtNAC072	AT4G27410			4.21	4.97
	DtNAC089	AT5G22290			5.42	
	DtNAC092	AT5G39610				1.44
	DtNAC101	AT5G62380				-3.32
	DtNAC105	AT5G66300		1.45		
NF-YA	*DtNF-YA7*	AT1G30500			3.05	3.99
	DtNF-YA2	AT3G05690			-1.45	
NF-YB	*DtNF-YB8*	AT2G37060				1.72
	DtNF-YB10	AT3G53340			1.56	2.02
	DtNF-YB3	AT4G14540				1.60
NF-YC	*DtNF-YC11*	AT3G12480				1.47
	DtNF-YC1	AT3G48590		1.43		1.54
	DtNF-YC4	AT5G63470				1.40
Nin-like	*DtNLP9*	AT3G59580				1.54
	DtNLP2 (2 contigs)	AT4G35270			-1.58	-1.69
RAV	*DtEDF3*	AT3G25730		1.70		
SBP	*DtSPL8*	AT1G02065			-3.90	
	DtSPL9	AT2G42200				-1.70
TALE	*DtBLH10*	AT1G19700			1.58	
	DtH1	AT4G32980		-3.98		-4.86
TCP	*DtTCP3*	AT1G53230				-1.77
	DtTCP4 (2 contigs)	AT3G15030			-2.75	-2.61
	DtTCP21	AT5G08330			-1.43	-2.71
Trihelix	*Dt-TF*	AT3G58630			1.55	
	DtVFP5	AT5G05550		1.50	-1.45	
	DtGT2L	AT5G28300				-2.08
WOX	*DtWOX4*	AT1G46480			-3.82	-5.42
WRKY	*DtWRKY6*	AT1G62300			-1.89	
	DtWRKY57	AT1G69310			1.61	
	DtWRKY25 (2 contigs)	AT2G30250		1.53		3.80
	DtWRKY46	AT2G46400				1.53
	DtWRKY75 (2 contigs)	AT5G13080			2.10	3.53
	DtWRKY74	AT5G28650			-2.46	-1.73
	DtWRKY27 (2 contigs)	AT5G52830			-1.64	
ZF-HD	*DtHB31*	AT1G14440			-2.29	-2.79
	DtHB32	AT1G14687			-2.54	-2.40
	DtHB29	AT1G69600			2.49	2.80
	DtHB21	AT2G02540				-1.58

5

-5

Supplementary Figure 5. Effect on transcription factor (TF) gene expression elicited by pre-harvest stress (S) and controls (C) at harvest (H) and after 7d of storage at 6 °C (P): heatmaps of log2 fold change of TFs showing Arabidopsis codes and gene names.

A Positively associated with cell death

gene name	AT code	CH vs SH	CP vs SP	CH vs CP	SH vs SP	
DtLMK1	AT1G07650			-1.80		Membrane proteins
DtL5	AT1G12290			-1.75	-1.75	
DtSOC3	AT1G17600			-3.65	-2.87	
DtTNL40 (2 contigs)	AT1G72840			-1.73	2.91	
DtEVR	AT2G31880			-1.86		
DtCRK13	AT4G23210			-4.70		
DtMSL10 (3 contigs)	AT5G12080			-2.54	-2.45	
DtMYB108	AT3G06490			3.15	2.70	TFs
DtNAC089	AT5G22290			5.42		
DtNAC092	AT5G39610			4.97	5.83	
DtALPHA-VPE	AT2G25940			2.30	2.69	proteases
DtGAMMA-VPE	AT4G32940			2.34		
DtCATHB2	AT1G02305			1.88	2.54	
DtCATHB3	AT4G01610			2.36	1.83	
DtMC2	AT4G25110				-1.40	
DtCEP1	AT5G50260			7.96	8.09	
DtVEP1	AT4G24220				1.45	other
DtAGD2 (3 contigs)	AT4G33680			-1.32	-2.77	
DtSTP13	AT5G26340				1.81	
DtOM66	AT3G50930			-3.55		
DtKTI1 (4 contigs)	AT1G73260		-0.91	6.81	4.09	

B Negatively associated with cell death

gene name	AT code	CH vs SH	CP vs SP	CH vs CP	SH vs SP
DtLEN1 (5 contigs	AT1G55490			-2.41	-2.30
DtRTP1	AT1G70260			-6.89	-6.27
DtRPI2	AT2G01290			-2.50	-1.85
DtMOD1 (3 contig	AT2G05990			-2.01	-2.24
DtFAH1 (2 contigs	AT2G34770			1.51	0.44
DtRAP2.3 (2 conti	AT3G16770			1.93	3.14
DtNRP2	AT3G27090			-1.80	
DtACD1	AT3G44880			2.83	2.65
DtSYP122	AT3G52400				-1.49
DtEDR3	AT3G60190				2.22
DtGFG1	AT4G12720			-2.43	-1.81
DtATILP	AT4G19700		1.78	2.57	5.34
DtFAH2	AT4G20870			-1.81	-2.69
DtERF109	AT4G34410				1.52
DtPCS1	AT5G02190			-2.28	-3.42
DtANN8	AT5G12380			1.52	1.96
DtNRP1	AT5G42050				1.72
DtETFBETA	AT5G43430				1.68
DtBI-1 (4 contigs)	AT5G47120			1.64	1.70

C Positively associated with senescence

gene name	AT code	CH vs SH	CP vs SP	CH vs CP	SH vs SP
DtNSH2	AT1G05620	-2.24			
Dt-GABA transporter	AT1G08230			5.26	4.95
DtBFN1	AT1G11190			8.61	10.12
Dt-senescence regulator	AT1G11700			-1.46	
DtCCS	AT1G12520			-4.77	-3.77
DtSRG1	AT1G17020			-1.51	
DtARP4	AT1G18450			-1.57	-2.30
Dt-senescence regulator (3 contigs)	AT1G29640			4.60	4.86
Dt-senescence regulator	AT1G61930			-2.25	-3.35
DtANAC029 (2 contigs)	AT1G69490	-1.70		5.18	6.40
DtSAG18	AT1G71190				2.64
DtOPR1 (2 contigs)	AT1G76680	1.57	-3.07	-2.01	-3.80
DtS40.4 (2 contigs)	AT2G28400			3.54	4.65
DtS40-1L (2 contigs)	AT2G34340	-1.69	-2.68	0.17	
DtYLS9	AT2G35980				2.57
DtORE9	AT2G42620				1.91
DtFLZ3 (3 contigs)	AT2G44670			1.65	0.97
DtCYP89A9	AT3G03470		-2.26	5.42	3.29
DtANAC046	AT3G04060			1.63	
DtDUF760-6	AT3G17800			1.49	
DtSAG21 (3 contigs)	AT4G02380		-2.24	2.45	3.81
DtWRKY42	AT4G04450				-2.45
Dt-senescence regulator	AT4G04630				2.98
DtPPDK (3 contigs)	AT4G15530			2.51	2.13
Dt-senescence associated	AT4G17670				-1.43
DtNCED4 (2 contigs)	AT4G19170		1.58		
Dt-senescence regulator	AT4G21930				2.58
DtSGR1 (3 contigs)	AT4G22920			2.68	2.49
DtSGR1	AT4G22920			-2.64	-2.86
Dt-senescence regulator	AT4G26950			-3.38	-4.20
DtSAG18	AT4G35770		2.34	4.47	6.45
Dt-senescence regulator (2 contigs)	AT5G03230	-1.93		2.15	2.89
DtPPH	AT5G13800			1.89	1.97
DtFTSH6	AT5G15250			12.23	12.38
Dt-senescence associated	AT5G20700	-1.42			1.47
DtCSAP	AT5G39520			8.15	8.00
DtANAC092	AT5G39610			4.97	5.83
DtCLH2	AT5G43860				-2.05
DtATS40-2	AT5G45630		1.57	6.31	8.70
DtSAG12	AT5G45890		-2.99	9.65	8.49
DtAPG7	AT5G45900			1.70	
DtPOAT7	AT5G51640			1.44	1.75
DtHP65B	AT5G55220			-1.70	
DtAOC2	AT3G25770		-2.99		-5.17

5

-5

D Negatively associated with senescence

gene name	AT code	CH vs SH	CP vs SP	CH vs CP	SH vs SP
DtPLGG1	AT1G32080			-6.34	-5.25
DtPES1	AT1G54570		-2.03	2.70	
DtCLE14	AT1G63245		1.84		2.24
DtRCA (9 contigs)	AT2G39730	1.57		-2.69	-2.73
DtPES2 (2 contigs)	AT3G26840-A			3.53	4.05
DtPES2 (3 contigs)	AT3G26840-B			-1.67	-1.71
DtPAD4	AT3G52430			-1.55	-2.15
DtMED28	AT3G52860				5.98
DtPP2-A13	AT3G61060		2.18		2.74
DtXDH1	AT4G34890			2.12	1.93
DtAPD7	AT5G02760				-1.93
DtETFbeta	AT5G43430				1.68
Dt-NEET	AT5G51720	1.96	-1.97		-3.07
DtLARP1B	AT5G66100			-2.88	-3.37

E autophagy-related

gene name	At code	SH vs CH	SP vs CP	CP vs CH	SPvs SH
DtAPG7	AT5G45900			1.70	
DtAPG8A (2 contigs)	AT4G21980			1.98	2.03
DtARAC9	AT2G44690			-2.88	-2.78
Dt-autophagy-like	AT5G16380				1.47
DtATG12A	AT1G54210			1.41	1.90
DtATG13	AT3G49590				1.51
DtATG8E (2 contigs)	AT2G45170			3.02	3.72
DtATG8F (2 contigs)	AT4G16520				1.60
DtATG8H	AT3G06420		-1.90	12.17	6.97
DtG18F	AT5G54730				1.91
DtHVA22D-a	AT4G24960				-1.44
DtHVA22D-b	AT4G24960			3.01	3.22
DtNBR1	AT4G24690				1.49
DtTSPO	AT2G47770			8.10	9.65
DtATI3A	AT1G17780				1.46
DtATG1A	AT3G61960				2.68
DtATG4A	AT2G44140			1.47	
DtUBAC2B	AT2G41160			3.97	3.73

Supplementary Figure 6. Response of positive (A,C) and negative (B,D) regulators of programmed cell death (PCD) and senescence, and (E), autophagy related in pre-harvest stress (S) and controls (C) at harvest (H) and after 7d of storage at 6 °C (P): heatmaps of log2 fold change showing Arabidopsis codes and gene names.

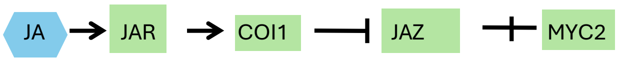

gene name	AT code	CH vs SH	CP vs SP	CH vs CP	SH vs SP
DtDAD1-activating factor (Activates JA biosynthesis)	AT5G05280			2.61	
DtLOX2 (JA biosynthesis) (5 contigs)	AT3G45140			-3.18	-4.26
DtAOC2 (JA biosynthesis)	AT3G25770		-2.99		-5.17
DtOPCL1 (2 contigs) (JA biosynthesis)	AT1G20510			-2.08	-0.58
DtACX1 (JA biosynthesis)	AT4G16760			1.50	1.45
DtOPR3 (JA biosynthesis)	AT2G06050				-1.80
JA biosynthesis (peroxisome)	AT4G05160			1.54	1.82
DtJMT (2 contigs) (MeJA from JA)	AT1G19640		-1.68		-2.30
DtJAZ1 (JA signaling)	AT1G19180				-3.53
DtJAZ2 (JA signaling)	AT1G74950				-2.90
DtJAZ8 (2 contigs)	AT1G70700		1.50	-3.82	-3.29
DtJAZ9 (JA signaling)	AT1G30135		-1.72		
DtJAZ10 (JA signaling)	AT5G13220		-2.08		
DtPR3 (JA signalling)	AT3G12500			-2.51	
DtMAT1 (affects JA signaling)	AT3G14395				-5.57
DtVIP1 (2 contigs) (JAZ co-receptor complex)	AT3G01310				-1.74
DtJUL1 (JA response) (2 contigs)	AT5G10650			1.72	1.75
DtMAPKKK14 (JA responsive)	AT2G30040				1.56
DtPDF1.2A (JA responsive)	AT5G44420			-1.47	-2.15
DtST2A (JA response) (2 contigs)	AT5G07010		-2.07	-3.77	-5.61
DtWRKY18	AT4G31800			-3.53	-3.55
DtWRKY33	AT2G38470		-1.74		
DtWRKY53	AT4G23810			-4.29	
DtWRKY54	AT2G40750		1.65	-4.44	-3.24

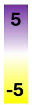

Supplementary Fig. 7. Pathway and Heat map (log2 fold change) of the DEGs related to jasmonic acid (JA) biosynthesis, signalling and response, including four JA-responsive WRKY transcription factors in stressed and control leaves at harvest (CH vs. SH) stressed and control leaves after 7 d of storage (CP vs. SP), control at harvest and after storage (CH vs. CS), and stressed leaves at harvest and after storage (SH vs. SP).

gene name	At code	CH vs SH	CP vs SP	CH vs CP	SH vs SP
DtGALK2	AT5G14470		3.68	4.29	8.80
Dt Ca-binding protein	AT1G21550		2.48		4.12
DtCML37	AT5G42380		2.33		1.77
DtZAT12	AT5G59820		1.77		3.86
DtATS40-2	AT5G45630		1.57	6.31	8.73
Dt DNAJ protein (3 contigs)	AT2G20560	-1.66	1.54	4.38	6.14
DtWRKY46 (2 contigs)	AT2G46400		1.48	-2.95	1.53
DtUGT73B3	AT4G34131		-1.64	5.21	3.12
Dt LSU3	AT3G49570		-3.44	2.31	

Supplementary Figure 8. Heat map (log2 fold change) of the DEGs between stressed and control leaves after 7 d of storage (CP vs. SP) that are also represented in the top 100 DEGs listed by Yamauchi et al. (2015) as responsive to treatment with 2-hexenal. Other DEG comparisons are: (CH vs. SH) stressed vs. control leaves at harvest, (CH vs. CP), control at harvest vs. after storage (SH vs. SP), stressed leaves at harvest vs. after storage.
