# Supplementary material for: Pre-harvest heat stress affects rocket salad leaf transcription and metabolism at harvest and after chilled postharvest storage
Source: Ann Bot. 2025 Nov 13:mcaf243. Online ahead of print. doi: 10.1093/aob/mcaf243 (PMC13737583; doi:10.1093/aob/mcaf243)
Supplement: Supplementary file 1 [file mcaf243_Supplementary_Data.zip › Alotaibi Supp Tables 1-4 FINAL REVISED.pdf]

Supplementary Table 1 list of contaminant VOCs excluded from the analysis

Common contaminants that were removed from the analysis as they are not of biological origin:

**Contaminant**	**Synomym 1**	**Synonym 2**
Acetonitrile		
Argon		
Benzene, trifluoromethyl		
Carbon dioxide		
Cyclopentasiloxane, decamethyl-		
Cyclotetrasiloxane, octamethyl-		
Cyclotrisiloxane, hexamethyl-		
Cyclodecasiloxane, eicosamethyl-		
Cyclotetrasiloxane, octamethyl-		
Dibutyl phthalate	1,2-Benzenedicarboxylic acid, dibutyl ester	1,2-Benzenedicarboxylate, dibutyl
Di-tert. Butyl phthalate	1,2-Benzenedicarboxylic acid, bis(2-methyl-propyl) ester	1,2-Benzenedicarboxylate, bis(2-methyl-propyl)
Diethyl Phthalate	1,2-Benzenedicarboxylic acid, di-ethyl ester	1,2-Benzenedicarboxylate, di-ethyl
Dimethyl phthalate	1,2-Benzenedicarboxylic acid, dimethyl ester	1,2-Benzenedicarboxylate, dimethyl
Dioctyl phthalate	1,2-Benzenedicarboxylic acid, bis(2-ethyl-hexyl) ester	1,2-Benzenedicarboxylate, bis(2-ethyl-hexyl)
Hydroxylamine		
Methylene chloride		
Nitrous oxide		
Propene		
Silane, dimethoxydimethyl-		
Silanediol, dimethyl-		
Sulfur dioxide		
Trichloromethane		

Supplementary Table 2-list of all primers used

primer name	primer sequence
DtEF1aF	TCTTGGTAGACGCCTTCACG
DtEF1aR	AGGAAGCGGTGTCATTGTTG
DtTCH4F	CCCCTGTCTCGAGCCATTAG
DtTCH4R	TTGGAACGCTGATGATTGGG
DtCRE1F	CACTCCTCGTACGTTGCATG
DtCRE1R	TGCTTTGGGTTTGCTTCAGA
DtPYL6F	GGGGTTCACAGGCTTCAGAA
DtPYL6R	AATTTCTCGGCAAGTTTCGC
DtPYL9F	ACTCTTCGATTGTGACACTTCA
DtPYL9R	CCAATCTTTCAGAAACATGTGCC
DtSAUR14F	TGCAAGGAATAGTGAGGCCA
DtSAUR14R	AGCAGATTCTCCGTCAAGCA
DtSAUR30F	AGAGTCCAAAACAGAGCCAGA
DtSAUR30R	CCAGCGTGTTTGAATCCGTA
DtSAUR72F	GACAAGAACGTGGCAAGGAA
DtSAUR72R	AATTCTCCCTCCGGTCTTCC
DtNAC019F	CGGGTTCAATGGTTTAGCGG
DtNAC01R9	GAGCAAAGCCAAAGTCGTCC
DtNAC059F	GCTCGGTTCATCATCGACT
DtNAC059R	CGAACCATCAAAACCGTGACC

Supplementary Table 3-list of VOCs and heatmap of VOC relative abundance

low – high

Compounds	Repetition 1	Repetition 2	mean RI	chemical family	IUPAC name	Repetition 1					Repetition 2			
						CPR1	SPR1	CHR1	SHR1		CPR1	SPR1	CHR1	SHR1
[1,1':3',1''-Terphenyl]-2'-ol	y	y	2193.5	aromatic	1,1':3',1''-Terphenyl-2'-ol	0.37	0.31	0.39	0.37		0.69	0.61	0.46	0.47
1,3-Cyclopentadiene, 1-methyl-	y	y	672.8	alkadiene	1-Methyl-1,3-cyclopentadiene	0.09	0.13	0.16	0.16		0.21	0.16	0.16	0.12
1-Butanol, 3-methyl-	y	y	742.8	alcohol	3-Methyl-1-butanol	0.16	0.18	0.13	0.14		0.12	0.12	0.09	0.11
1-Hexanol	y	y	871.1	alcohol	1-Hexanol	1.21	1.72	1.88	1.83		0.64	0.63	1.14	1.14
1-Hexanol, 2-ethyl-	y	y	1032.2	alcohol	2-Ethyl-1-hexanol	0.37	0.37	0.40	0.40		0.48	0.49	0.42	0.40
1-Octadecanol	y	y	2068.2	alcohol	1-Octadecanol	0.38	0.37	0.34	0.37		0.44	0.46	0.27	0.36
1-Octanol	y	y	1074.2	alcohol	1-Octanol	0.16	0.15	0.20	0.15		0.13	0.14	0.11	0.11
1-Penten-3-ol	y	y	699.0	alcohol	1-penten-3-ol	2.21	2.56	2.70	2.54		1.75	2.05	2.23	2.39
1-Penten-3-one NS1	y	y	701.8	ketone	1-Penten-3-one	0.91	1.22	1.06	0.87		0.61	1.34	0.68	1.34
2(5H)-Furanone, 5-ethyl- (?)	y	y	985.7	lactone	5-Ethyl-2(5H)-furanone	1.20	1.54	0.97	0.43		0.55	2.44	0.59	2.50
2,4-Hexadienal	y	y	920.1	aldehyde	2,4-hexadienal	0.27	0.33	0.21	0.12		0.18	0.31	0.18	0.26
2-Decenal	y	y	1273.0	aldehyde	2-Decenal	0.26	0.26	0.23	0.23		0.27	0.31	0.19	0.27
2-Heptanone	y	y	894.1	ketone	2-Heptanone	0.23	0.22	0.25	0.26		0.30	0.26	0.24	0.21
2-Hexen-1-ol, (E)-	y	y	868.3	alcohol	(2E)-2-hexen-1-ol	1.00	1.61	1.41	1.59		0.58	0.80	0.90	1.12
2-Hexen-1-ol, acetate	y	y	1016.3	ester	(2E)-2-Hexen-1-yl acetate	1.36	1.29	1.41	1.49		0.88	0.56	1.28	0.99
2-Hexenal	y	y	852.6	aldehyde	2-Hexenal	0.21	0.25	0.15	0.14		0.14	0.23	0.13	0.26
2-Hexenal, (E)-	y	y	736.1	aldehyde	(E)-2-hexenal	0.20	0.24	0.25	0.21		0.18	0.17	0.14	0.19
2-Nonenal	y	y	1170.2	aldehyde	2-Nonenal	0.21	0.18	0.19	0.19		0.25	0.25	0.16	0.20
2-Pentanone	y	y	702.0	ketone	2-Pentanone	0.26	0.31	0.30	0.31		0.42	0.36	0.30	0.30
2-Penten-1-ol, (E)-	y	y	771.6	alcohol	2-Penten-1-ol	0.82	1.06	1.14	0.99		0.37	0.40	0.59	0.61
2-Penten-1-ol, (Z)-	y	y	774.1	alcohol	(2Z)-2-Penten-1-ol	0.60	0.61	0.52	0.43		0.30	0.49	0.31	0.47
2-Penten-1-ol, acetate	y	y	913.4	ester	2-Penten-1-yl acetate	1.13	0.68	0.91	0.76		0.47	0.34	0.62	0.49
2-Pentenal	y	y	764.2	aldehyde	2-pentenal	0.55	0.71	0.56	0.44		0.32	0.71	0.38	0.84
2-Pentene, 1-ethoxy-4-methyl	y	y	1239.5	ether	1-Ethoxy-4-methyl-2-pentene	0.48	0.41	0.23	0.24		0.24	0.39	0.17	0.40
3-Carene	y	y	1019.7	terpene	3-carene	0.16	0.13	0.14	0.16		0.20	0.20	0.14	0.19
3-Ethyl-1,5-octadiene	y	y	946.7	alkadiene	3-Ethyl-1,5-octadiene	0.24	0.24	0.28	0.22		0.17	0.15	0.23	0.19
3-Hexen-1-ol, (E)-	y	y	854.0	alcohol	(E)-3-hexenol	0.56	0.81	0.65	0.66		0.42	0.54	0.49	0.66
3-Hexen-1-ol, (Z)-	y	y	860.1	alcohol	(3Z)-3-Hexen-1-ol	3.59	3.76	3.83	4.25		4.83	4.55	4.22	3.96
3-Hexen-1-ol, acetate, (E)-	y	y	1007.9	ester	(3E)-3-hexen-1-yl acetate	4.84	4.38	4.66	5.30		6.29	4.92	5.89	4.80
3-Hexen-1-ol, acetate, (Z)-	y	y	1003.9	ester	(3Z)-3-hexen-1-yl acetate	1.46	1.24	1.27	1.15		0.77	0.55	1.18	1.01
3-Hexen-1-ol, propanoate	y	y	1100.3	ester	3-Hexen-1-yl propionate	1.57	1.58	1.10	1.03		0.48	0.72	0.76	0.76

3-Hexenal	y	y	803.5	aldehyde	3-Hexenal	1.80	2.38	1.91	1.18	0.53	3.58	1.41	2.78
3-Hexenoic acid, methyl ester	y	y	933.4	ester	Methyl -3-hexenoate	0.22	0.24	0.25	0.25	0.21	0.21	0.19	0.19
3-Methylhexyl isothiocyanate	y	y	1284.9	isothiocyanate	1-Isothiocyanato-3-methylhexane	0.23	0.24	0.23	0.23	0.23	0.25	0.18	0.21
3-Pentanol	y	y	713.3	alcohol	3-Pentanol	0.47	0.49	0.62	0.48	0.43	0.30	0.52	0.43
3-Pentanone	y	y	709.8	ketone	3-Pentanone	1.91	2.22	2.26	2.11	1.04	1.13	1.52	1.55
4-Hexen-1-ol, acetate	y	y	1026.7	ester	(4E)-4-Hexen-1-yl acetate	0.77	0.61	0.78	0.76	0.50	0.29	0.67	0.46
4-Methylpentyl isothiocyanate	y	y	1173.8	isothiocyanate	1-isothiocyanato-4-methylpentane	1.00	1.45	0.83	1.18	0.57	0.85	0.52	0.78
Acetic acid, hexyl ester	y	y	1013.3	ester	Hexyl acetate	2.24	1.92	2.46	2.34	1.42	0.66	2.21	1.53
Acetic acid, pentyl ester	y	y	915.0	ester	Pentyl acetate	0.48	0.36	0.49	0.43	0.28	0.20	0.38	0.21
Acetoin	y	y	722.0	ketone	3-Hydroxy-2-butanone	0.41	0.19	0.21	0.21	0.23	0.21	0.16	0.22
Acetophenone	y	y	1084.5	ketone	1-Phenylethanone	0.61	0.54	0.72	0.55	1.03	0.90	0.49	0.77
Aldehyde	y	y	1049.2	aldehyde	Aldehyde	0.81	0.92	0.63	0.38	0.37	0.98	0.45	1.01
Alkadiene	y	y	998.7	alkadiene	Alkadiene	0.39	0.38	0.41	0.34	0.33	0.35	0.34	0.36
Alkanal1	y	y	1111.6	aldehyde	Aldehyde	0.39	0.36	0.53	0.35	0.41	0.41	0.29	0.36
Alkane-3	y	y	1320.0	alkane	Alkane	0.22	0.21	0.19	0.23	0.21	0.24	0.16	0.20
Alkane-35	y	y	1273.6	alkane	Alkane	0.28	0.32	0.26	0.26	0.29	0.40	0.21	0.36
Alkane-60	y	y	1021.8	alkane	Alkane	0.43	0.40	0.48	0.50	0.37	0.45	0.41	0.60
Alkane-83	y	y	1600.1	alkane	Alkane	0.37	0.32	0.39	0.37	0.41	0.35	0.27	0.26
Alkene191212	y	y	2103.0	alkene	Alkene	0.34	0.37	0.40	0.41	0.53	0.61	0.35	0.45
Alkynes	y	y	919.2	alkyne	Alkyne	0.18	0.22	0.14	0.09	0.12	0.31	0.11	0.36
Benzaldehyde	y	y	979.2	aromatic	Benzaldehyde	0.58	0.54	0.60	0.59	0.85	0.78	0.52	0.60
Benzene, 1,2,3-trimethyl-	y	y	1011.8	aromatic	1,2,3-Trimethylbenzene	0.13	0.12	0.17	0.16	0.20	0.20	0.19	0.16
Benzene, 1,3-dimethyl-	y	y	878.5	aromatic	m-Xylene	0.31	0.38	0.42	0.42	0.29	0.30	0.29	0.26
Benzene, 1-ethyl-4-methyl-	y	y	971.0	aromatic	1-Ethyl-4-methylbenzene	0.15	0.15	0.22	0.20	0.16	0.17	0.15	0.14
BRANCHED ALKANE 12	y	y	1314.6	alkane	Alkane	0.21	0.19	0.20	0.25	0.25	0.21	0.18	0.16
Butanoic acid, 3-hexenyl ester	y	y	1188.5	ester	(3E)-3-Hexen-1-yl butyrate	2.59	1.98	1.44	1.37	0.88	0.75	1.34	0.94
C2-alkyl -benzene	y	y	869.7	aromatic	alkyl-benzene	0.24	0.29	0.31	0.32	0.26	0.26	0.28	0.26
Decanal	y	y	1213.7	aldehyde	Decanal	0.39	0.39	0.55	0.39	0.35	0.33	0.29	0.30
Decane	y	y	1000.1	alkane	Decane	0.25	0.23	0.30	0.25	0.27	0.30	0.23	0.26
D-Limonene	y	y	1040.5	terpene	D-limonene	0.25	0.22	0.28	0.33	0.21	0.33	0.23	0.28
Dodecanal	y	y	1417.8	aldehyde	Lauraldehyde	0.29	0.27	0.31	0.27	0.30	0.27	0.22	0.21
Dodecane	y	y	1200.0	alkane	Dodecane	0.32	0.30	0.34	0.30	0.33	0.39	0.29	0.34
Ester	y	y	1144.7	ester	ester	1.17	0.95	0.51	0.50	0.36	0.85	0.45	0.71

Ester-10	y	y	832.1	ester	ester	0.22	0.22	0.24	0.25	0.22	0.20	0.19	0.21
Ester-7	y	y	923.8	ester	ester	0.36	0.37	0.41	0.31	0.30	0.29	0.34	0.31
Eucalyptol	y	y	1046.6	terpene	1,3,3-Trimethyl-2-oxabicyclo[2.2.2]octane	0.33	0.30	0.40	0.46	0.25	0.26	0.31	0.24
Formamide, N-methyl-	y	y	713.2	amide	N-Methylformamide	0.47	0.49	0.62	0.48	0.44	0.31	0.53	0.43
Furan, 2-ethyl-	y	y	711.9	furan	2-Ethylfuran	0.45	0.58	0.44	0.36	0.36	0.76	0.41	0.69
Furan, 2-pentyl-	y	y	995.2	furan	2-Pentylfuran	0.28	0.26	0.24	0.24	0.27	0.33	0.18	0.29
Heptanal	y	y	907.9	aldehyde	Heptanal	0.39	0.36	0.39	0.42	0.42	0.38	0.32	0.29
Heptane	y	y	709.3	alkane	Heptane	0.44	0.43	0.45	0.41	0.30	0.27	0.22	0.24
Hexanal	y	y	805.7	aldehyde	Hexanal	0.39	0.60	0.63	0.45	0.16	0.60	0.37	0.71
Hexane, 1-isothiocyanato-	y	y	1211.8	isothiocyanate	1-isothiocyanatohexane	0.51	0.79	0.48	0.56	0.37	0.47	0.41	0.50
Hexane, 1-nitro-	y	y	1055.0	nitroalkane	1-Nitrohexane	0.33	0.35	0.31	0.32	0.23	0.56	0.25	0.54
Hexanoic acid	y	y	974.2	carboxylic acid	Hexanoic acid	0.20	0.19	0.22	0.14	0.21	0.31	0.10	0.44
Hexanoic acid, 2-oxo-, methyl ester	y	y	1047.8	ester	Methyl 2-oxohexanoate	0.30	0.37	0.27	0.20	0.20	0.44	0.20	0.35
Hexanoic acid, 3-hexenyl ester, (Z)-	y	y	1384.3	ester	(3Z)-3-Hexen-1-yl hexanoate	0.30	0.19	0.18	0.15	0.17	0.15	0.15	0.14
Methyl formate	y	y	581.7	ester	Methyl formate	0.44	0.35	0.35	0.41	0.61	0.84	0.53	0.89
n-Nonylcyclohexane	y	y	1567.7	alkane	Nonylcyclohexane	0.15	0.14	0.15	0.16	0.15	0.17	0.11	0.13
Nonane	y	y	900.0	alkane	Nonane	0.28	0.26	0.32	0.31	0.28	0.24	0.24	0.19
n-Pentyl isothiocyanate	y	y	1108.7	isothiocyanate	1-isothiocyanatopentane	0.41	0.59	0.37	0.44	0.28	0.34	0.28	0.32
n-Valeric acid cis-3-hexenyl ester	y	y	1234.7	ester	(Z)-3-Hexenyl valerate	1.46	1.38	0.57	0.58	0.44	1.08	0.33	0.99
Octanal	y	y	1009.9	aldehyde	Octanal	2.65	2.02	2.97	2.47	2.75	1.40	3.76	2.22
Octane	y	y	799.9	alkane	Octane	0.37	0.27	0.33	0.43	0.16	0.22	0.22	0.18
Pentadecane	y	y	1500.1	alkane	Pentadecane	0.34	0.32	0.35	0.35	0.31	0.30	0.24	0.22
Phenol	y	y	983.5	aromatic	Phenol	0.49	0.50	0.52	0.48	0.79	0.69	0.42	0.58
Tetradecane	y	y	1400.0	alkane	Tetradecane	0.39	0.38	0.38	0.40	0.35	0.34	0.28	0.26
Thiocyanic acid, methyl ester	y	y	726.0	sulphur compound	methyl thiocyanate	0.65	1.12	0.58	0.83	0.49	0.81	0.42	0.65
Thiophene, 2,5-dihydro-	y	y	774.5	sulphur compound	2,5-Dihydrothiophene	0.19	0.22	0.21	0.17	0.17	0.22	0.19	0.20
Thiophene, ethyl-	y	y	874.8	sulphur compound	2-Ethylthiophene	0.20	0.26	0.17	0.18	0.13	0.23	0.16	0.31
Thiophene, tetrahydro-	y	y	818.7	sulphur compound	Tetrahydrothiophene	0.18	0.27	0.30	0.27	0.21	0.26	0.22	0.19
Tridecane	y	y	1300.1	alkane	Tridecane	0.32	0.29	0.30	0.31	0.28	0.29	0.23	0.24
Tridecane, 2-methyl-	y	y	1364.1	alkane	2-Methyltridecane	0.19	0.18	0.19	0.18	0.19	0.21	0.12	0.17
Tridecane, 3-methyl-	y	y	1371.3	alkane	3-Methyltridecane	0.30	0.23	0.24	0.26	0.18	0.19	0.14	0.15
Undecanal	y	y	1315.7	aldehyde	Undecanal	0.24	0.22	0.27	0.24	0.28	0.25	0.20	0.19
Undecane	y	y	1100.0	alkane	Undecane	1.57	1.58	1.10	1.03	0.17	0.26	0.16	0.20

	VOCs detected in the two repetitions				
**Compounds**	**Repetition 1**	**Repetition 2**	**mean RI**	**chemical family**	**IUPAC name**
(1S)-2,6,6-Trimethylbicyclo[3.1.1]hept-2-ene	y		943.3	terpene	(1S,5S)-2,6,6-Trimethylbicyclo[3.1.1]hept-2-ene
2-Ethyl-1-dodecanol	y		1493.6	alcohol	2-Ethyl-1-dodecanol
2-Ethylhexyl salicylate	y		1827.5	ester	2-Ethylhexyl salicylate
2-n-Butyl furan	y		895.5	furan	2-Butylfuran
2-Undecenal	y		1376.2	aldehyde	2-Undecenal
Acetic acid, 2-ethylhexyl ester	y		1148.2	ester	2-Ethylhexyl acetate
Acetic acid, butyl ester	y		816.0	ester	Butyl acetate
Acetic acid, methyl ester	y		615.1	ester	Methyl acetate
Alcohol-1	y		1309.5	alcohol	alcohol
Alcohol-2	y		1480.7	alcohol	alcohol
Alkane-5	y		1454.7	alkane	Alkane
Alkanol-1 (alcohol)	y		1333.1	alcohol	alcohol
Disulfide, dimethyl	y		756.1	sulphur compound	(Methyldisulfanyl)methane
Dodecane, 2,6,10-trimethyl-	y		1375.2	alkane	2,6,10-Trimethyldodecane
Dodecane, 4,6-dimethyl-	y		1255.3	alkane	4,6-Dimethyldodecane
Ester-1	y		1387.2	ester	ester
Heptadecane	y		1700.2	alkane	Heptadecane
Octadecane	y		1800.1	alkane	Octadecane
Pentadecane, 2-methyl-	y		1563.9	alkane	2-Methylpentadecane
Propane, 1-isothiocyanato-3-(methylthio)-	y		1336.4	isothiocyanate	1-isothiocyanato-3-(methylsulfanyl)propane
Tetradecanal	y		1622.4	aldehyde	Myristaldehyde
Tetradecane, 3-methyl-	y		1471.3	alkane	3-Methyltetradecane
Thiolanes	y		1021.6	sulphur compound	sulphur compound
Toluene	y		775.9	aromatic	Toluene

	VOCs detected in the two repetitions				
Compounds	Repetition 1	Repetition 2	mean RI	chemical family	IUPAC name
2(3H)-Furanone, dihydro-5-pentyl-		y	1380.9	lactone	5-Pentyldihydro-2(3H)-furanone
2,3-Butanediol		y	691.7	alcohol	2,3-Butanediol
Alkene-7		y	902.8	alkene	Alkene
Benzoic acid		y	1163.3	carboxylic acid	Benzoic acid
Benzoic acid, 2-methylpropyl ester		y	1346.5	ester	Isobutyl benzoate
Benzothiazole		y	1259.4	sulphur compound	1,3-Benzothiazole
Carbon disulfide		y	621.3	sulphur compound	Methanedithione
Eicosane		y	2000.1	alkane	Eicosane
Heneicosane		y	2072.2	alkane	Henicosane
Nonanoic acid		y	1261.7	carboxylic acid	Nonanoic acid
Pentanoic acid, 2,2,4-trimethyl-3-carboxyisopropyl, isobutyl ester		y	1597.0	ester	1-Isobutyl 4-isopropyl 3-isopropyl-2,2-dimethylsuccinate
Phenylmaleic anhydride		y	1569.6	furanone	3-Phenyl-2,5-furandione
Undecane, 4,8-dimethyl-		y	1261.3	alkane	4,8-Dimethylundecane
Unknown2		y	651.4	unknown	unknown

Supplementary Table 4 – RNAseq data

Sample	RawReads	FinalReads	Stress	Time
NSP.r1_S7	37,065,061	27,556,931	Non-stressed	Post-harvest
NSP.r2_S8	37,597,989	27,979,446	Non-stressed	Post-harvest
NS.r1_S1	36,289,122	27,229,407	Non-stressed	Harvest
NS.r2_S2	37,655,291	28,477,892	Non-stressed	Harvest
NS.r3_S3	42,276,692	32,435,064	Non-stressed	Harvest
SNP.r3_S9	41,022,265	30,963,126	Non-stressed	Post-harvest
SP.r1_S10	40,873,357	30,871,387	Stressed	Post-harvest
SP.r2_S11	40,257,327	30,416,183	Stressed	Post-harvest
SP.r3_S12	37,364,823	28,068,789	Stressed	Post-harvest
S.r1_S4	39,287,230	30,055,687	Stressed	Harvest
S.r2_S5	39,169,589	30,172,312	Stressed	Harvest
S.r3_S6	37,606,372	28,819,094	Stressed	Harvest
